# Mechanistic insights into the nickel-dependent allosteric response of the *Helicobacter pylori* NikR transcription factor

**DOI:** 10.1016/j.jbc.2022.102785

**Published:** 2022-12-09

**Authors:** Karina A. Baksh, Jerry Augustine, Adnan Sljoka, R. Scott Prosser, Deborah B. Zamble

**Affiliations:** 1 Department of Biochemistry, University of Toronto, Toronto, Ontario, Canada; 2 Department of Chemistry, University of Toronto, Toronto, Ontario, Canada; 3 RIKEN Center for Advanced Intelligence Project, RIKEN, Chuo-ku, Tokyo, Japan

**Keywords:** allosteric regulation, metalloprotein, transcription factor, DNA-binding protein, DNA-protein interaction, bacterial transcription, nickel, structure-function, computational biology, metal homeostasis, DBD, DNA-binding domain, DTNB, 5,5′-dithiobis-(2-nitrobenzoic acid), FA, fluorescence anisotropy, GGS, gly-gly-ser, MBD, metal-binding domain, RTA, rigidity-transmission allostery

## Abstract

In *Helicobacter pylori*, the nickel-responsive NikR transcription factor plays a key role in regulating intracellular nickel concentrations, which is an essential process for survival of this pathogen in the acidic human stomach. Nickel binding to *H. pylori* NikR (HpNikR) allosterically activates DNA binding to target promoters encoding genes involved in nickel homeostasis and acid adaptation, to either activate or repress their transcription. We previously showed that HpNikR adopts an equilibrium between an open conformation and DNA-binding competent *cis* and *trans* states. Nickel binding slows down conformational exchange between these states and shifts the equilibrium toward the binding-competent states. The protein then becomes stabilized in a *cis* conformation upon binding the *ureA* promoter. Here, we investigate how nickel binding creates this response and how it is transmitted to the DNA-binding domains. Through mutagenesis, DNA-binding studies, and computational methods, the allosteric response to nickel was found to be propagated from the nickel-binding sites to the DNA-binding domains *via* the β-sheets of the metal-binding domain and a network of residues at the inter-domain interface. Our computational results suggest that nickel binding increases protein rigidity to slow down the conformational exchange. A thymine base in the *ureA* promoter sequence, known to be critical for high affinity DNA binding by HpNikR, was also found to be important for the allosteric response, while a modified version of this promoter further highlighted the importance of the DNA sequence in modulating the response. Collectively, our results provide insights into regulation of a key protein for *H. pylori* survival.

Many transition metals are essential trace nutrients for living organisms due to their role as cofactors or regulators (1, 2, 3). Organisms devote significant resources to maintain a sufficient supply of these metals (4); however, despite being indispensable, high concentrations are cytotoxic (5, 6). Maintaining the balance between metal starvation and toxicity is particularly challenging for bacterial pathogens, especially those facing host defense mechanisms (7, 8, 9). Bacteria tightly regulate metal bioavailability through metal-responsive transcription factors, also known as metalloregulators or metal-sensing proteins. These metal-responsive transcription factors detect the bioavailability of a specific type of metal in the cell and subsequently regulate the transcription of genes encoding proteins that control the acquisition, storage, delivery, and efflux pathways of that metal (10, 11, 12, 13, 14, 15, 16). Metalloregulators are usually allosterically regulated by the metal ion for which they are responsible, such that metal binding to specific sites on the protein influences DNA binding at a distal region (10). A wide variety of mechanisms have been proposed for allosteric regulation of these metal-sensing proteins, ranging from large structural changes to subtle changes in dynamics (10, 13). However, the details of the allosteric mechanism of regulation of the Ni(II)-responsive NikR transcription factor from *Helicobacter pylori* remains elusive despite its central role in maintaining nickel homeostasis.

*H. pylori* colonization of the human stomach causes various gastrointestinal diseases, including gastric cancer (17, 18, 19). Nickel is an essential nutrient for this bacterium because it serves as a cofactor for the urease enzyme, which neutralizes the intracellular pH, permitting the survival of *H. pylori* within the fluctuating pH conditions of the human stomach (20, 21, 22, 23). Nickel is also the cofactor for the [NiFe]-hydrogenase enzyme that allows *H. pylori* to use hydrogen gas produced by other gut bacteria as an energy source (24, 25, 26). The NikR metalloregulator is key to maintaining nickel homeostasis in *H. pylori* because it regulates the transcription of various genes encoding proteins involved in maintaining nickel homeostasis, as well as acid adaptation, such as the urease enzyme precursor proteins (27, 28, 29, 30, 31). NikR senses the bioavailability of nickel, which allosterically activates high affinity DNA binding and complex formation with the promoters of target genes (10, 32, 33, 34, 35). *H. pylori* NikR (HpNikR) has a more complex activity than other known NikR proteins because it functions as either a transcriptional activator or repressor (36), binding with a wide range of affinities to its various target sequences (37, 38) that share a weak consensus (39, 40).

NikR is an obligate homotetramer with a central C-terminal metal-binding domain (MBD) connected by flexible linkers to two peripheral N-terminal DNA-binding domains (DBDs) that adopt a ribbon-helix-helix fold (Fig. 1) (41, 42, 43, 44, 45, 46, 47, 48, 49, 50). In solution, NikR proteins bind nickel with high affinity (32, 51, 52, 53, 54), and in crystal structures of HpNikR and its homologs in *Escherichia coli* (EcNikR) and *Pyrococcus horikoshii* (PhNikR), four identical and conserved square planar His_3_Cys nickel sites were observed per tetramer (43, 46, 47, 48, 49, 55). These sites are also observed in the crystal structure of the Ni(II)–HpNikR–DNA complex (Fig. 1) (56); however, crystal structures of Ni(II)-HpNikR in the absence of DNA have been reported that show additional coordination sites besides the square planar sites, which are thought to be due to differences in crystallization and purification conditions (42, 44, 45).

**Figure 1:**
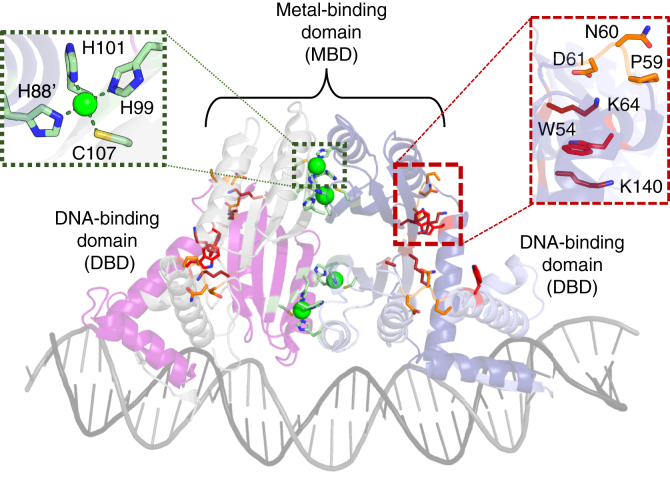
**Crystal structure of Ni(II)-HpNikR-DNA (PDB:****6MRJ****).** Each protomer is shaded in a different color, and the DNA is shown in *gray*. Nickel ions (*green spheres*) are coordinated in a square planar geometry by His99, His101, Cys107 from one monomer and His88′ from an opposing monomer (*green sticks*; shown in *dotted line* inset) in the metal-binding domain. Residues of interest at the inter-domain interface (*red sticks*) and inter-domain linkers (*orange sticks*) that were chosen for mutagenesis are shown in the *dashed line* inset.

NikR proteins adopt different conformational states—designated as open, *trans*, and *cis*—based on the positions of the DBDs with respect to the MBD, as shown in the various crystal structures of HpNikR, EcNikR, and PhNikR (42, 46, 47, 48, 49). In the open state, the DBDs extend outwards, whereas for the *cis* and *trans* conformers, the DBDs flank the tetramer, oriented towards opposite sides of the tetramer for the *trans* state, or the same side of the tetramer for the *cis* state. The *cis* state has been observed in the crystal structures of EcNikR and HpNikR in complex with nickel and DNA (48, 56); however, the *trans* state is also likely to be DNA-binding compatible and serve as an intermediate state for DNA binding (35). Using ^19^F-NMR, we recently found that apo-HpNikR undergoes rapid interconversion between the open state and the two DNA-binding compatible (*cis* and *trans*) states. Nickel-binding shifts the conformational equilibrium toward the DNA-binding competent states, while also reducing the mobility of the DBDs (35). We found that in the presence of the *ureA* promoter, a well-studied target DNA sequence (30, 32, 33, 34, 37, 57, 58), HpNikR becomes stabilized in a slightly asymmetric *cis* conformation, in a manner consistent with conformational selection (35). However, it remains unclear how the allosteric signal is propagated from the nickel-binding sites in the MBD to the DBDs to create this response. HpNikR has two unique structural features compared to its homologs through which the allosteric signal induced by nickel might be transmitted—its longer inter-domain linkers and a nonconserved tryptophan at the MBD/DBD interface that is important for Ni(II)-mediated DNA recognition (44, 45, 50, 59). Our current knowledge of the allosteric mechanisms of metal-regulated DNA binding is not yet comprehensive, but in some metalloregulators, the allosteric response is transmitted through changes in a H-bonding network upon metal binding (10), while in others, a metal induced redistribution of internal dynamics occurs, which was only recently uncovered (10, 60, 61). In general, the concept of allostery driven by changes in dynamics—in the absence of clear-cut conformational transformations—has only been more recently applied to metalloregulators, but none larger than a homodimer (10). Although studies have speculated on the allosteric mechanisms of HpNikR, no clear model has emerged (42, 50, 58).

To determine the mechanism of the Ni(II)-dependent allosteric response in HpNikR, we used mutagenesis, DNA-binding studies, and computational studies to investigate pathways through which the allosteric signal is transmitted from the nickel-binding sites to the DBDs. Nickel binding was found to produce a long-range effect in HpNikR, with the allosteric response propagated from the nickel-binding residues through the MBD β-sheets to Lys64 and Lys140 at the MBD/DBD interface, either of which forms a cation-π interaction with Trp54 on the DBDs. The computational analysis revealed that the response to nickel might involve an increase in rigidity throughout the protein, which slows down the conformational exchange. In addition, the analysis indicated that allosteric transmission through HpNikR occurs asymmetrically. Accordingly, a high level of allosteric transmission was predicted to a critical thymine on one half site of the HpNikR recognition sequence in the *ureA* promoter (38), signifying its importance for the two-step DNA-binding process (57) that ultimately results in the protein being stabilized in a slightly asymmetric *cis* conformation upon complex formation (35). Fluorescence anisotropy experiments to examine DNA-binding with a modified version of the promoter (named *ureA*-perf) (35, 37) further emphasized the important role the DNA sequence plays in tuning the allosteric response of HpNikR, which contributes to our understanding of how HpNikR is able to regulate such a wide variety of genes. These results provide novel insights into how HpNikR responds to nickel and DNA, leading to an updated model of allosteric regulation of HpNikR. The knowledge gained for HpNikR advances our overall understanding of mechanisms of allosteric control, particularly in cases where conformational changes resulting from ligand binding are subtle.

## Results

### Trp54 at the MBD/DBD interface is essential for allosteric response

**Figure 2:**
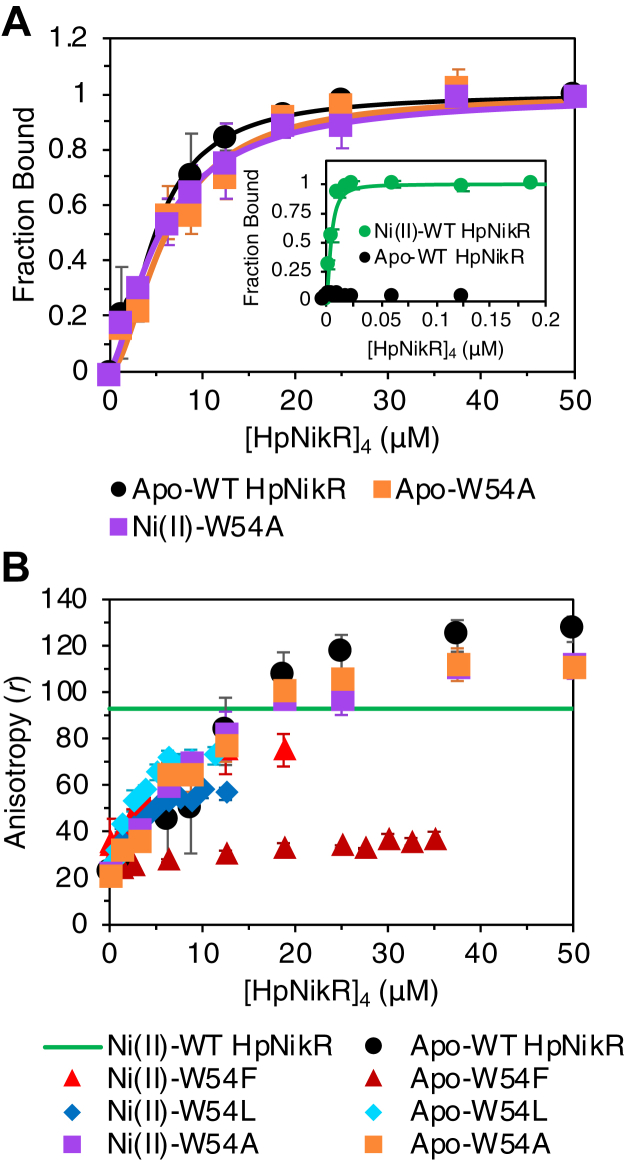
**Fluorescence anisotropy-monitored DNA binding of WT HpNikR and Trp54 mutants with the ureA promoter**. *A*, apo-WT and apo- and Ni(II)-W54A. The data from each replicate were fit to the Hill equation, and the calculated DNA-binding affinities are shown in Table 1. Inset: fluorescence anisotropy-monitored binding of apo- and Ni(II)-WT binding the ureA promoter. *B*, change in anisotropy, r, of apo- and Ni(II)-WT HpNikR, W54A, W54F, and W54L. Ni(II)-HpNikR is shown as a line to indicate the values it saturates at, which is in a lower concentration range, as shown in the inset from (A). The anisotropy values of apo- and Ni(II)-bound W54F and W54L are smaller than that of apo- and Ni(II)-WT HpNikR at saturation upon binding the ureA promoter. Experiments were performed with 5 nM of the ureA promoter in 3 mM MgSO4, 20 mM Tris, 100 mM NaCl, pH 7.6. The data points represent the average derived from the preparation of three samples at each protein concentration, and the error bars represent ± one SD.

To learn more about the mechanism by which the allosteric response is transmitted from the nickel binding sites to the DBDs, mutagenesis studies were performed to identify key residues participating in the allosteric response. HpNikR has a sole, nonconserved, tryptophan (Trp54) located on the DBDs at the MBD/DBD interface (Fig. 1), which was previously found to abrogate Ni(II)-activated DNA binding when mutated to alanine (44). It was originally postulated that Trp54 plays a role in positioning the DBD with respect to the MBD in order to form a stable complex with the HpNikR DNA recognition sequence when the protein is Ni(II)-bound (44, 45). Due to its location at the inter-domain interface, it is likely that Trp54 also plays a role in transmitting the allosteric response produced by nickel binding. To investigate this possibility, several mutants were created, and the allosteric coupling free energy (Δ*G*_c_) was calculated to examine how allosteric regulation was affected in each mutant (62). For HpNikR, Δ*G*_c_ is the quantitative measure of the extent to which DNA binding is positively coupled to nickel binding (10, 62, 63, 64) and is calculated from the DNA-binding affinities of the apo- and Ni(II)-bound proteins with a 32 bp oligonucleotide containing the HpNikR recognition sequence from the *ureA* promoter (35). In this case, reliable binding isotherms of the *ureA* promoter to HpNikR are obtained by monitoring the fluorescence anisotropy (FA) of fluorescein-tagged DNA as a function of concentration of the HpNikR tetramer (Fig. 2*A*) (35). None of the mutants exhibited altered Ni(II)-binding activity or disrupted secondary structure compared to WT HpNikR (Figs. S1 and S2, Table S1).

The affinity of apo-W54A for the *ureA* promoter is approximately 1-fold weaker than that of apo-WT HpNikR, whereas Ni(II)-W54A binds the *ureA* promoter three orders of magnitude weaker than Ni(II)-WT (Table 1 and Fig. 2*A*). Thus, the resulting Δ*G*_c_ is only −0.1 ± 0.07 kcal/mol for W54A, compared to a Δ*G*_c_ of −4.1 ± 0.2 kcal/mol for WT HpNikR, indicating that nickel binding and DNA binding are uncoupled in W54A (Table 2). In addition, the similar affinities of Ni(II)-W54A, apo-W54A, and apo-WT HpNikR for the *ureA* promoter demonstrates that nickel can no longer activate high affinity DNA binding without Trp54. The anisotropies of the fluorescein-tagged *ureA* promoter in the presence of apo- and Ni(II)-W54A saturate around similar values as apo-WT HpNikR, as shown in Figure 2*B*, which are higher than that of Ni(II)-HpNikR, further supporting that the removal of the tryptophan side chain causes DNA binding by the Ni(II)-bound protein to be similar to that of the apo-protein. Different anisotropy values upon saturation for apo- *versus* Ni(II)-HpNikR are not unexpected, as ^19^F-NMR previously showed that the DNA-bound complex formed by apo-HpNikR is somewhat different (35), which could result in the fluorophore in the *ureA* promoter sequence to be buried differently upon binding. These results underlie the essential role of Trp54 for transmitting the allosteric signal created upon nickel binding.

**Table 1:** DNA-binding affinities determined by FA experiments with the *ureA* promoter

HpNikR	*K*_d, apo_ (M) × 10^−6^	*n*	*K*_d, Ni(II)-bound_ (M) × 10^−9^	*n*
WT	4.9 ± 1.7	1.8 ± 0.4	4.5 ± 0.6	1.8 ± 0.4
W54A	6.1 ± 1.0	1.7 ± 0.2	5379.0 ± 110.0	1.4 ± 0.2
W54F	N/A	N/A	N/A	N/A
W54L	N/A	N/A	N/A	N/A
K64A	N/A	N/A	22.0 ± 2.2	1.4 ± 0.2
K140A	N/A	N/A	63.0 ± 5.6	1.4 ± 0.1
K64A/K10A	N/A	N/A	N/A	N/A
K64M/K140M	N/A	N/A	N/A	N/A
Δ3aa	N/A	N/A	4.4 ± 1.3	1.8 ± 0.3
GGS	N/A	N/A	4.6 ± 1.2	2.1 ± 0.8

N/A – *K*_d_ not determined due to saturation at lower raw anisotropy values than WT HpNikR.

To examine the importance of the side chain aromaticity for the allosteric response, a W54F mutation was created. While apo- and Ni(II)-W54F elicited an effect in the FA experiment, indicating that DNA binding was occurring, the anisotropy values were significantly lower at saturation than for WT HpNikR, especially for apo-W54F (Fig. 2*B*). This result indicates that replacing tryptophan with phenylalanine affects the DNA-bound complex with or without nickel. Although the values for Ni(II)-W54F are closer to Ni(II)-WT than apo-W54F and apo-WT, saturation occurs at lower concentrations for Ni(II)-W54F compared to Ni(II)-WT (high μM *versus* low nM, respectively). EMSA results for Ni(II)-W54F using the same 32 bp *ureA* promoter sequence (without the fluorescein label) showed a DNA-bound band with an identical shift as WT HpNikR—indicating the formation of similarly sized DNA-bound complexes—but the free DNA band contained smears that were not present in the WT HpNikR controls run on the same gel (Fig. S3, *A* and *B*). The presence of smears for the free DNA, but not the DNA-bound band, indicates a difficulty in ability to form the same DNA-bound complex as WT HpNikR, resulting in multiple smaller DNA-bound complexes being formed instead. The EMSA results are consistent with the FA results that suggest a different DNA-bound complex is being formed based on the lower anisotropy values at saturation. EMSAs were not performed for apo proteins due to protein aggregation during the assay at the concentrations required for observable DNA binding.

A W54L mutant created to replace the Trp54 with another hydrophobic residue also produced similar results as W54F in the FA experiment, showing lower anisotropy values at saturation (Fig. 2*B*). Therefore, it appears that the tryptophan side chain is required for key interactions at the MBD/DBD interface for the Ni(II)-bound protein that cannot occur with a different aromatic or hydrophobic side chain. However, in the absence of nickel, the tryptophan side chain does not seem to be essential for DNA binding, as indicated by the FA results of apo-W54A. It is interesting to note that although DNA binding is impaired for both mutants, Ni(II)-W54F saturates at higher anisotropy values than apo-W54F, whereas apo-W54L saturates at higher values than Ni(II)-W54L. Two lysine residues from the MBD (Lys64 and Lys140) are situated around Trp54, seemingly forming a pocket around it (Fig. 1) (35, 44, 45). It is possible that mutating the tryptophan residue to leucine or phenylalanine affects the interaction with the two lysine residues, thereby disturbing the pocket, and the ability to properly bind DNA. The leucine side chain might be small enough that it does not disrupt the pocket as much as phenylalanine in the apo protein but is unable to form the interaction with the lysine residues required for Ni(II)-responsive binding. The higher anisotropy values for Ni(II)-W54F than those for apo-W54F suggests that the phenylalanine might either be able to form the correct interaction but the interface is still disrupted or that it is better able to fit into the pocket when the protein is Ni(II)-bound. Accordingly, there is a slight decrease in α-helicity for apo-W54F compared to apo-WT or the other Trp54 mutants (Fig. S2 and Table S1). The results of these mutants overall indicate that the allosteric signal induced by nickel binding is transmitted to Trp54 on the DBDs.

### Probing the interaction between Trp54 and two MBD lysine residues

**Figure 3:**
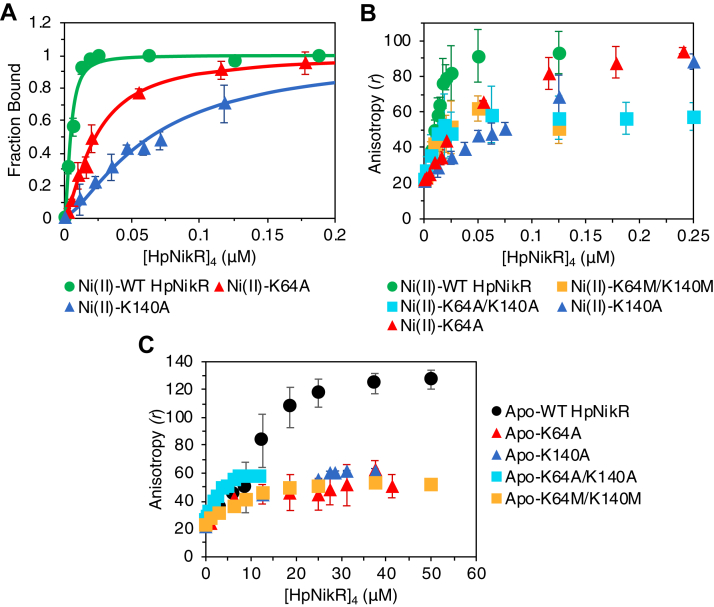
**Fluorescence anisotropy-monitored DNA binding of WTHpNikRand lysine mutantswith the *ureA* promoter.***A*, Ni(II)-K64A and Ni(II)-K140A bind DNA weaker than Ni(II)-WT. Calculated DNA-binding affinities are shown in Table 1. *B*, change in anisotropy, *r*, of Ni(II)-K64A/K140A and Ni(II)-K64M/K140M are lower than that of Ni(II)-WT HpNikR binding to DNA. *C*, change in anisotropy, *r*, of all of the apo-lysine mutants are lower than that of apo-WT HpNikR. Experiments and data processing were done as described in Figure 2.

To investigate whether the MBD lysine residues interact with Trp54 and are involved in allosteric transmission, Lys64 and Lys140 were first individually mutated to alanine. FA experiments of Ni(II)-bound K64A and K140A revealed that their DNA-binding affinities to the *ureA* promoter were respectively 4- and 11-fold weaker than Ni(II)-WT HpNikR (Table 1 and Fig. 3*A*). Although the impact of mutating these lysine residues is not as large as when Trp54 is mutated, the weakened DNA-binding affinities suggest they interact with Trp54 to enable allosteric transmission.

It is possible that that the two lysine residues do not act as a pair and that only one is required for the Ni(II)-activated DNA-binding activity of HpNikR. To test this possibility, a K64A/K140A double mutant was created. Just as was observed for W54F, EMSAs performed with Ni(II)-K64A/K140A contained smears that did not appear for WT HpNikR (Fig. S3), and the FA results showed saturation at lower anisotropy values (Fig. 3*B*). These results suggest that the interaction between at least one of the two lysine residues and Trp54 is sufficient for allosteric transmission upon nickel binding as well as the ability to properly bind DNA, but both lysine residues are required for optimal binding.

The nature of the interaction between the lysine residues and Trp54 was further probed with a K64M/K140M double mutant to remove the positive charge of the lysine side chain while mimicking its length. The results for Ni(II)-K64M/K140M mirrored that of the double alanine mutant, indicating that the cation is the key component for the Trp54 interaction when the protein is nickel bound (Fig. 3*B*). These results suggest a cation-π interaction with tryptophan arises from either of the two lysine residues in Ni(II)-HpNikR. The abnormal DNA-binding exhibited by W54F indicates that the cation-π interaction with tryptophan is stronger than with phenylalanine, which has been observed before in other proteins (65). Alternatively, it is possible that phenylalanine is unable to properly fit into the pocket created by the lysine side chains, affecting the formation of a DNA-bound complex regardless of being able to form the cation-π interaction.

**Figure 4:**
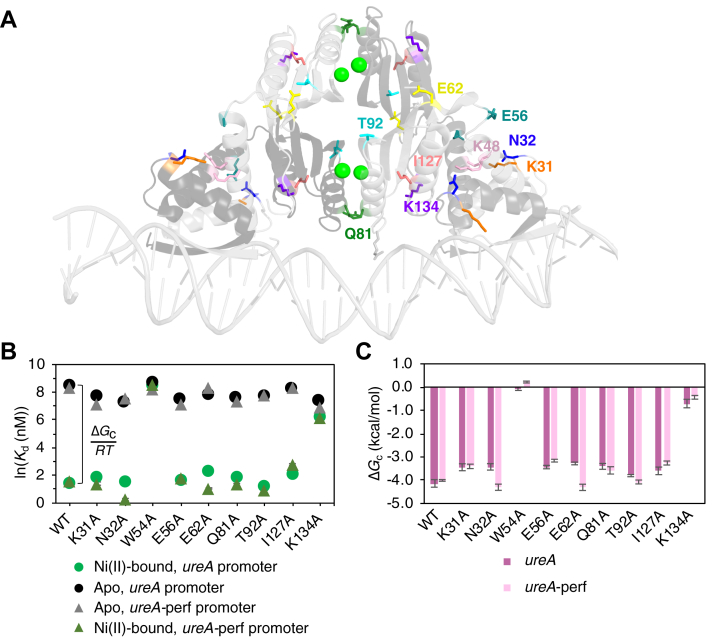
**DNA-binding activity and allosteric coupling of HpNikR mutans chosen from rigidity-transmission allostery (RTA) analysis.***A*, crystal structure of Ni(II)-HpNikR-DNA (PDB:6MRJ) indicating residues chosen for mutagenesis based on the RTA analysis, which are labeled on one monomer. Nickel ions are shown as *green spheres*. *B*, DNA-binding affinities (*K*_d_) for WT and mutant HpNikR proteins bound to the *ureA* promoter (*circles*) or the *ureA*-perf promoter (*triangles*) when apo (*black*/*gray*) or Ni(II)-bound (*green*/*light green*), as shown in Table 2. *C*, graphical representation of the allosteric coupling free energy (Δ*G*_c_) values of mutants chosen from the RTA analysis binding the *ureA* promoter *versus* the *ureA*-perf promoter, as shown in Table 2.

In the absence of nickel, all of the lysine mutants reached saturation at lower anisotropy values than WT HpNikR (Fig. 4*C*), indicating that removal of either Lys64 or Lys140 impairs DNA binding in the apo protein. Taken together with the similar DNA-binding affinities of apo-W54A compared to apo-WT, it appears that both lysine residues—but not Trp54—are needed for the apo protein to properly bind DNA. This indicates that the cation-π interaction is part of the allosteric response to Ni(II)-binding and is not essential for the ability of the protein to bind DNA in the absence of nickel. Instead, due to the already very weak DNA-binding affinity, any disruptions to the pocket created by the lysine side chains seems to impair the ability of the apo-protein to form the conformation required for proper DNA-binding.

### The allosteric signal is not transmitted through the MBD/DBD linkers

Since the inter-domain linkers are required to facilitate conformational exchange between the open, *cis*, and *trans* states, they are also a likely route for relaying the allosteric response upon nickel binding (59). The inter-domain linkers in HpNikR are slightly longer than those in its homologs, which is thought to contribute to its unique binding response (Fig. S4) (50). To investigate the role of the length of the linkers, three nonconserved residues (Pro59, Asn60, Asp61) were removed to create a Δ3aa mutant. The role of increased linker flexibility was also examined by substituting these residues (Pro59, Asn60, Asp61) to Gly-Gly-Ser (GGS) (66). Surprisingly, both mutants had similar DNA-binding activity as WT HpNikR when loaded with nickel, whereas in the absence of nickel, the anisotropies saturated at lower values than the WT protein, similar to the results of the apo-lysine mutants (Table 1 and Fig. S5).

The lack of an impact by these mutants on DNA-binding in the presence of nickel indicates that shorter or more flexible linkers do not affect sampling of the DNA-binding competent states or complex formation. It is possible that the allosteric response to nickel binding compensates for any changes to the linkers. For the apo-protein, changing the linker might also change the position of the DBDs in a way that makes it more difficult to contact the HpNikR recognition sequence, since DNA binding in the absence of nickel is already very weak. Overall, it appears that the allosteric response upon nickel binding is transmitted from the Ni(II)-binding residues to the lysine residues and Trp54 at the MBD/DBD interface rather than *via* the inter-domain linkers.

### Searching for additional allosteric residues in HpNikR

To learn more about allosteric networks of residues or pathways that may connect the nickel binding residues to Lys64 and Lys140 at the MBD/DBD interface, rigidity-transmission allostery (RTA) computational analysis was performed (67, 68). RTA methods utilize mathematical rigidity theory and graph algorithms (69, 70, 71) to analyze allosteric networks within protein structures (72, 73). This approach provides a mechanistic view of allostery by analyzing how local perturbation of rigidity and conformational degrees of freedom propagates from one site to modify rigidity at distant sites across the protein. Starting with a high-resolution experimental structure, we first generate a constraint network (graph), where the protein is viewed in terms of vertices (atoms) and edges (*i.e.*, covalent bonds, electrostatic bonds, hydrogen bonds, and hydrophobic contacts). Hydrogen bonds are ranked in terms of overall energy strength according to local donor-hydrogen-acceptor geometry. RTA then measures transmission (changes) of degrees of freedom across all residues in protein structure as a consequence of rigidification of a distant site.

The His_3_Cys nickel-binding sites that are conserved among NikR homologs are also present in crystal structures of HpNikR, including the structure of the protein in complex with nickel and DNA; however, additional sites with unclear biological relevance have been observed in some structures of HpNikR (42, 43, 44, 45, 46, 47, 48, 49, 55). Thus, we chose to rigidify the DNA instead of nickel as a starting point to probe possible allosteric networks. Since there is no publication accompanying the Ni(II)-HpNikR-DNA structure, we chose to begin our analysis using the crystal structure of EcNikR in complex with nickel and DNA because there is more information available about it (48, 56). Furthermore, in the crystal structure of Ni(II)-HpNikR-DNA, the N-terminal extension, which is absent in HpNikR homologs (Fig. S4), is not resolved. This region plays an important role in DNA-binding affinity and specificity of HpNikR and is thought to be loosely structured and able to contact the DNA (74, 75, 76). Examining allosteric pathways of HpNikR from the DNA sequence in the absence of its N-terminal extension could therefore complicate the interpretation. HpNikR and EcNikR share 30% identity but 68% similarity (30), which is high enough to make reasonable comparisons between the two, and although HpNikR binds several target promoters, we would expect similar allosteric principles in response to nickel for either protein. Thus, structure of Ni(II)-EcNikR-DNA was chosen for this analysis, and changes in rigidity across the protein structure were monitored upon rigidifying the DNA (Fig. S6). This analysis was used as a starting point to identify residues for mutagenesis that could be potentially allosteric, which could then be further examined *via* RTA analysis in the Ni(II)-HpNikR-DNA structure. The RTA analysis revealed several allosteric residues in EcNikR that are in communication with the DNA. Promisingly, a sequence alignment of HpNikR and EcNikR revealed that the residue that corresponds to Trp54 in EcNikR is allosteric. To select residues for a mutagenesis study in HpNikR based on the RTA analysis of EcNikR, a Sneath’s index analysis was performed, which assesses the dissimilarity between amino acids based on 134 categories of activity and structure (77). Nine residues in HpNikR were chosen for mutagenesis due to their high similarity to the corresponding residues in EcNikR (Fig. 4*A*). None of the mutants exhibited altered Ni(II)-binding activity or disrupted secondary structure compared to WT HpNikR (Fig. S7 and Table S2).

Allosteric coupling between nickel and the *ureA* promoter is largely affected in K134A, for which the allosteric free coupling energy is reduced to ∼17% of that of the WT protein (Table 2). Interestingly, all of the apo-mutants exhibit tighter DNA-binding affinities for the *ureA* promoter than apo-WT HpNikR (Table 2 and Fig. S8). However, for most of the mutants, the Δ*G*_c_ is ∼80% of that of the WT protein, indicating there is only a small impact on allosteric coupling with this promoter (Table 2). Surprisingly, in the FA experiment, K48A exhibited no DNA binding in the absence or presence of nickel. It was previously determined in another study that Lys48 forms an inter-subunit salt bridge with Glu47 when Ni(II)-HpNikR is bound to the *ureA* promoter; EMSAs showed a 60-fold reduction in the DNA-binding affinity of Ni(II)-K48A compared to Ni(II)-WT HpNikR (75). Differences observed in Ni(II)-K48A binding to the *ureA* promoter are possibly due to the EMSA running conditions stabilizing interactions between the protein and DNA through a caging effect, restricting diffusion and promoting binding (78). The lack of DNA binding determined here by FA for K48A, regardless of nickel, indicates that the salt bridge with Glu47 is essential for binding to the *ureA* promoter, but it is unclear whether this salt bridge is important for allostery or only for the formation of the DNA-bound complex. Nevertheless, the Lys134 residue that showed the largest effect on allosteric regulation when mutated to alanine is located close to the MBD/DBD interface on the same β-strand as Lys140 and could potentially be part of the same allosteric network as Lys64/Lys140 and Trp54.

**Table 2:** DNA-binding affinities and coupling free energies for WT and mutant HpNikR proteins (identified from RTA analysis) with the *ureA* or *ureA*-perf promoter as determined by FA experiments

Promoter	HpNikR	*K*_d, apo_ (M) × 10^−6^	*n*	*K*_d, Ni(II)-bound_ (M) × 10^−9^	*n*	Δ*G*_c_ (kcal/mol)
*ureA*	WT	4.9 ± 1.7	1.8 ± 0.4	4.5 ± 0.6	1.8 ± 0.4	−4.1 ± 0.2
	W54A	6.1 ± 1.0	1.7 ± 0.2	5379.0 ± 110.0	1.4 ± 0.2	−0.1 ± 0.07
	K31A	2.3 ± 0.5	2.0 ± 0.2	6.8 ± 1.0	2.3 ± 0.2	−3.4 ± 0.1
	N32A	1.6 ± 0.4	1.1 ± 0.2	4.8 ± 0.9	1.6 ± 0.2	−3.4 ± 0.1
	K48A	No binding	N/A	No binding	N/A	N/A
	E56A	1.8 ± 0.3	1.8 ± 0.3	5.4 ± 0.1	1.9 ± 0.1	−3.4 ± 0.1
	E62A	2.6 ± 0.2	1.8 ± 0.2	10.1 ± 1.1	1.5 ± 0.1	−3.3 ± 0.1
	Q81A	2.0 ± 0.5	1.4 ± 0.2	6.5 ± 1.0	1.3 ± 0.3	−3.4 ± 0.1
	T92A	2.2 ± 0.2	2.0 ± 0.1	3.6 ± 0.2	1.5 ± 0.1	−3.8 ± 0.05
	I127A	4.0 ± 1.3	2.4 ± 0.3	8.7 ± 1.3	2.4 ± 0.3	−3.6 ± 0.2
	K134A	1.7 ± 0.7	1.6 ± 0.4	515.0 ± 40.0	1.0 ± 0.1	−0.7 ± 0.2
*ureA*-perf	WT	4.1 ± 1.1	1.8 ± 0.2	4.7 ± 0.4	1.9 ± 0.2	−4.0 ± 0.04
	W54A	3.7 ± 0.2	1.6 ± 0.1	5336.0 ± 500.0	1.8 ± 0.3	0.2 ± 0.04
	K31A	1.3 ± 0.2	1.6 ± 0.4	4.1 ± 1.0	1.5 ± 0.2	−3.4 ± 0.1
	N32A	1.9 ± 0.7	1.0 ± 0.4	1.3 ± 0.4	1.6 ± 0.2	−4.3 ± 0.1
	K48A	No binding	N/A	No binding	N/A	N/A
	E56A	1.2 ± 0.1	1.5 ± 0.2	5.9 ± 0.8	1.7 ± 0.1	−3.2 ± 0.1
	E62A	4.2 ± 0.7	1.5 ± 0.2	2.9 ± 0.9	1.4 ± 0.3	−4.3 ± 0.1
	Q81A	1.6 ± 0.6	1.4 ± 0.1	3.9 ± 1.4	1.4 ± 0.2	−3.6 ± 0.2
	T92A	2.5 ± 0.4	2.0 ± 0.1	2.6 ± 0.5	1.2 ± 0.2	−4.1 ± 0.1
	I127A	4.0 ± 0.3	1.4 ± 0.3	16.2 ± 3.2	1.2 ± 0.1	−3.3 ± 0.1
	K134A	1.0 ± 0.3	1.0 ± 0.2	491.0 ± 90.0	1.0 ± 0.1	−0.4 ± 0.1

### Allosteric regulation in HpNikR is also modulated by the DNA sequence

HpNikR has a larger regulon than EcNikR and binds several target promoters—that have a weak consensus sequence—with a wide range of affinities (37, 38, 39, 40). It is possible that there are differences in the allosteric response depending on the DNA sequence to allow HpNikR to bind these promoters. Therefore, it is likely that the residues chosen from the RTA analysis as being in communication with the DNA in EcNikR could play a role in the DNA-binding activity of HpNikR with other DNA sequences. To test this possibility, the Δ*G*_c_ for the mutants chosen from the RTA analysis was determined when binding a modified version of the *ureA* promoter (named *ureA*-perf) that was previously used in ^19^F-NMR studies (35). The HpNikR recognition sequence consists of two half-sites that are a pseudo-symmetric palindrome (37). The *ureA*-perf promoter is a perfectly symmetric version of the *ureA* promoter for the half site containing a thymine known to be crucial for tight DNA binding (38). ^19^F-NMR revealed that HpNikR binds both *ureA* and *ureA*-perf promoters with similar affinity, but with slightly different *cis* conformations (35).

As observed with the *ureA* promoter, many of the mutants bind *ureA*-perf tighter in the apo state than apo-WT HpNikR; however, an increase in the affinity for *ureA*-perf was also observed in the presence of nickel for several mutants (Table 2; Figs. 4*B* and S9). As a result, the magnitude of Δ*G*_c_ for some of the mutants are greater than or similar to WT HpNikR when using the *ureA*-perf promoter (Fig. 4*C*). This is quite different from the results with the *ureA* promoter, which showed a lower magnitude in Δ*G*_c_ for all mutants than the WT protein (Table 2). In the case of N32A and E62A, and to a lesser extent T92A, the magnitude of Δ*G*_c_ is greater than it is for WT HpNikR, indicating that binding nickel and binding this symmetric promoter is more tightly coupled when any of these side chains are removed. Surprisingly, the Δ*G*_c_ for W54A is positive with *ureA*-perf (Δ*G*_c_ = 0.2 ± 0.04 kcal/mol); the low value indicates very weak coupling but nevertheless suggests this mutant is slightly more likely to bind this DNA sequence in the absence of nickel. As with the *ureA* promoter, K48A exhibits no DNA binding to *ureA*-perf.

The differences in allosteric coupling observed for these mutants with the symmetric *ureA*-perf promoter compared to the pseudo-symmetric *ureA* promoter supports the model that the DNA sequence plays a role in tuning the allosteric response of HpNikR. The variable changes in the magnitude of Δ*G*_c_ for the mutants with the *ureA*-perf promoter compared to the overall decrease in Δ*G*_c_ with the *ureA* promoter indicates that the residues involved in the allosteric transmission pathways also differ depending on the DNA sequence, which implies they could differ depending on the target DNA promoter.

### Allosteric pathways from Trp54 and Lys134

**Figure 5:**
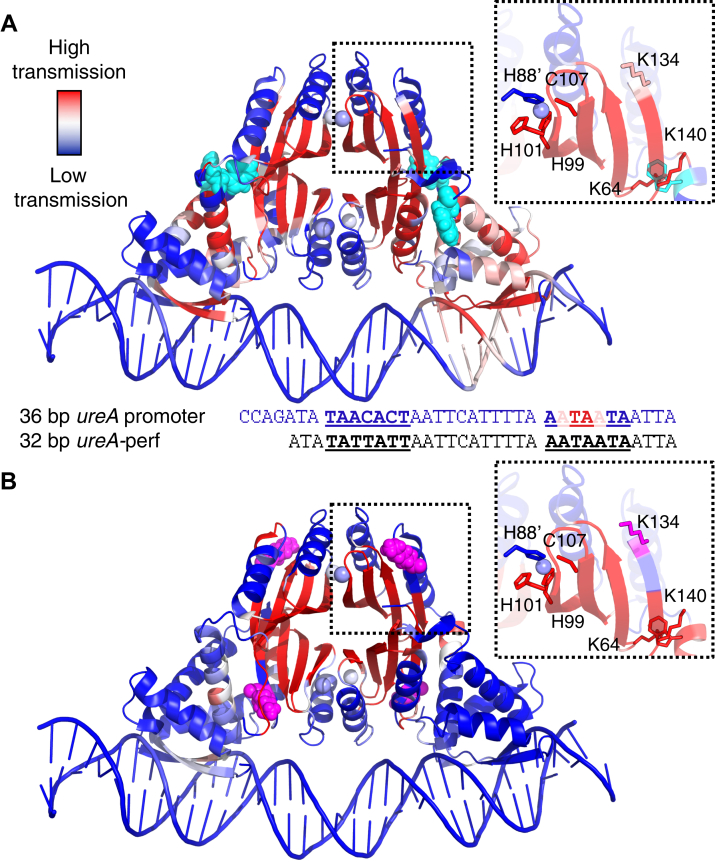
**Crystal structure ofNi(II)-HpNikR-DNA(PDB:****6MRJ****) showing allosteric transmission pathways predicted by rigidity theory analysis.** In *A*, Trp54 (*cyan spheres*) was rigidfied, and in *B*, Lys134 (*pink spheres*) was rigidified. Crystal structures are colored representing the magnitude of allosteric transmission from the specified residue to the rest of the protein. Inset shows a section of the MBD with important residues for allosteric transmission, as determined by mutagenesis and DNA-binding studies. Sequence under (*A*) corresponds to the 36 bp *ureA* promoter sequence shown in the crystal structure with the HpNikR recognition sequence underlined. Underneath is the 32 bp *ureA*-perf sequence used for FA experiments. FA, fluorescence anisotropy; MBD, metal binding domain.

Mutagenesis studies showed that Trp54 and Lys134 are important allosteric residues in HpNikR. Therefore, RTA analyses were performed rigidifying these residues in the crystal structure of Ni(II)-HpNikR bound to the *ureA* promoter in order to identify possible allosteric pathways connecting these two residues to the nickel sites (Fig. 5). The transmission pathway involving Trp54 extends from the nickel sites to the DBDs and onto the DNA, whereas the pathway involving Lys134 is confined to the MBD. This analysis is consistent with the larger change in Δ*G*_c_ for W54A than K134A (∼2% *versus* ∼17% of the WT protein, respectively).

As shown in Figure 5, the analyses for both Trp54 and Lys134 show high allosteric transmission to three of the four Ni(II)-binding residues (His101, His99, and Cys107) in square planar His_3_Cys sites, which are conserved among NikR homologs (28). Due to the symmetric nature of allosteric communication wherein the signal transmits in both directions between two sites, this result indicates that allosteric transmission from the nickel-binding residues is propagated to Trp54 and Lys134 at the MBD/DBD interface. Nickel is also coordinated by His88 from another monomer to make up the square planar site, but low transmission to that residue is observed from Trp54 and Lys134. In both analyses, there is also high transmission to Lys64 and Lys140, which is consistent with the mutagenesis studies showing that they are involved in the allosteric response through their interaction with Trp54 (Fig. S10). There appears to be some asymmetry in how the allosteric signal is propagated through the protein, as evidenced by the higher level of transmission observed in one DBD than the other in the Trp54 analysis. In the Lys134 analysis, high transmission to Trp54 is only shown on one monomer in that same DBD, whereas there is high transmission to Lys134 in three of the four monomers in the Trp54 analysis. In addition, the residues mutated in the inter-domain linker for Δ3aa and GGS show low allosteric transmission in both analyses, except for one monomer of the Trp54 analysis, which is consistent with the lack of an impact observed by FA for these mutants on Ni(II)-activated DNA binding. Therefore, the RTA analysis rigidifying Trp54 and Lys134 supports the model derived from the experimental data that Trp54, Lys134, Lys64, and Lys140 are all part of the allosteric network linking the nickel sites and the DBDs.

In the analyses of both Trp54 and Lys134, the regions showing high allosteric transmission in the MBD look very similar and primarily involve the residues in the β-sheets, which further supports that Trp54 and Lys134 are part of the same allosteric network (Fig. 5). These results indicate that the allosteric response is transmitted from the nickel-binding residues to Lys64/Lys140 through residues in the MBD β-sheets. From the mutants created based on the RTA analysis from EcNikR, only Thr92 and Glu62 are found in these β-sheets (Fig. S11). Although some monomers in either analysis show high levels of transmission to these two residues, both of them are found on the edges of the β-sheets beside areas of low transmission, which is likely why mutating them to alanine did not largely affect allosteric coupling between nickel and DNA. It is also possible that the deletion of these residues can be compensated by other residues, such that allosteric coupling is only minimally affected. Interestingly, as mentioned above, the magnitude of Δ*G*_c_ is slightly greater with the *ureA*-perf promoter than with the *ureA* promoter for both these mutants, suggesting that they might be more important for the allosteric response to different promoter sequences, as has been previously observed (75). High transmission is also observed for K48A on the DBDs, in accordance with its known importance for DNA binding (75). The remaining residues that were chosen for mutagenesis based on the EcNikR RTA analysis show low allosteric transmission, consistent with the small effect on Δ*G*_c_ when they were mutated to alanine, validating the experimental conclusion that they are not part of the allosteric network upon nickel binding.

In the Trp54 analysis, the DBD showing higher allosteric transmission is bound to a region of the DNA also showing high transmission, which maps to thymine at position 10 and adenine at position 11 of the HpNikR recognition sequence in the *ureA* promoter (Fig. 5*A*). The thymine at position 10 is only found on one half site and is known to be critical for tight DNA binding (38), as mentioned above. Our previous ^19^F-NMR results indicate Ni(II)-HpNikR adopts a slightly asymmetric *cis* conformation upon binding the *ureA* promoter, which was hypothesized to be due to this thymine (35). The higher level of transmission to the thymine and the DBD bound to it supports the hypothesis that the thymine is involved in the DNA-binding process for Ni(II)-bound HpNikR with the *ureA* promoter.

### Examining differences in rigidity between apo and Ni(II)-HpNikR

Our mutagenesis studies and RTA analyses indicate that the response to nickel is transmitted to the DBDs through an allosteric network of residues linked to the nickel sites. To learn more about how nickel binding is able to slow down the exchange between the open, *cis*, and *trans* states (35), we performed computational mathematical rigidity theory analysis on the apo- and Ni(II)-bound HpNikR crystal structures to probe the effect of nickel on overall structural stability. The apo- and Ni(II)-bound crystal structures published by Dian *et al.* (42) were used for this analysis. The Ni(II)-bound crystal structure by Dian *et al.* (42) shows six nickel ions overall; two are coordinated to the square planar His_3_Cys sites, two are coordinated to “intermediate” sites thought to be involved in nickel transport to the aforementioned sites, while the final two are coordinated to “external” sites. We note that the allosteric responses of metalloregulators are believed to be dependent on metal coordination (10, 79, 80, 81, 82). There are two crystal structures of Ni(II)-HpNikR that show only the four, conserved, square planar His_3_Cys sites—as observed in the Ni(II)-HpNikR-DNA crystal structure. However, one of the structures only has the MBD resolved (43), and the other is a mutant that exhibits weakened DNA binding (44). Therefore, the structures by Dian *et al.* (42) are the only ones that allow for a comparison of the effect of nickel on the full-length protein. Although the biological relevance of the additional nickel sites is unclear, it has been suggested that the method of preparing Ni(II)-bound HpNikR can affect nickel coordination (44). The method used by Dian *et al.* (42), which involves adding nickel to the apo protein, is consistent with how Ni(II)-HpNikR is prepared in our studies, including our ^19^F-NMR (35). Furthermore, nickel titration results in a subsequent study by the same group are consistent with our results shown in Figs. S1*B* and S7*D* (41). Therefore, the crystal structure was used to examine how nickel binding might affect HpNikR rigidity.

The method FIRST, which uses mathematical rigidity theory to analyze protein flexibility, was used to decompose HpNikR into flexible and rigid clusters (69, 71). As shown in Fig. S12, distinct rigid clusters are designated by different colors while flexible connections are shown in gray. Ni(II)-bound HpNikR is dominated by one large rigid cluster (shown in red) that persists over a large range of energy cutoffs in the hydrogen bond dilution plot, indicating high stability. In contrast, most of the apo-protein breaks into several rigid clusters at a lower hydrogen bond energy cutoff of −1 kcal/mol.

Interestingly, for both the apo- and Ni(II)-bound proteins, the MBD in chain A has higher rigidity (shown by red blocks in the hydrogen bond dilution plot), than in chain B. However, there is an overall increase in protein rigidity when HpNikR is bound to nickel, since both chains of the Ni(II)-HpNikR decompose into rigid clusters at higher hydrogen bond energy cutoffs than the apo-protein. Furthermore, the DBDs of both chains are highly rigid for Ni(II)-HpNikR, which is not the case for either chain of apo-HpNikR. The increased rigidity observed beyond the nickel-binding sites in the protein indicates that the mechanistic allosteric response upon nickel binding might involve rigidification. This increase in rigidity is consistent with the decrease in DBD mobility as previously observed by ^19^F-NMR and activation of high affinity DNA binding in the presence of nickel (35).

## Discussion

HpNikR is a Ni(II)-responsive pleiotropic transcriptional regulator of various genes in *H. pylori* that encode proteins involved in nickel homeostasis and acid adaptation, which are important for colonization and survival in the host (27, 28, 29, 30, 31). Due to its extensive responsibilities and the relatively low number of annotated transcription factors in the *H. pylori* genome compared with similar bacteria (83, 84, 85), HpNikR is considered to be a “master regulator” in *H. pylori* (28). Upon binding nickel, complex formation with target DNA promoters is allosterically activated. We previously determined using ^19^F-NMR that the apo-protein rapidly samples the open and DNA-binding competent *cis*/*trans* states. Upon binding nickel, the conformational equilibrium is shifted towards the *cis*/*trans* states, while the DBD mobility is reduced (35). Upon binding the *ureA* promoter, Ni(II)-HpNikR becomes stabilized in a slightly asymmetric *cis* conformation (35). Here, we sought to determine how the allosteric response upon nickel binding is transmitted through the protein to activate this response. Using mutagenesis, DNA-binding studies, and computational studies, we found that the allosteric effect upon nickel binding is propagated from the nickel-binding residues through the MBD β-beta sheets and to DBDs using an allosteric network of residues involving Trp54, Lys140, Lys64, and Lys134 at the MBD/DBD interface. Nickel binding also appears to cause an increase in protein rigidity, which would effectively slow down the DBD mobility and conformational exchange.

Trp54 at the MBD/DBD interface was previously investigated using a W54A mutant to examine its role in Ni(II)-responsive DNA recognition (44). In that study, no binding was observed to the *ureA* promoter with up to 300 nM of Ni(II)-W54A, implying that Trp54 played an important role in orienting the DBDs with respect to the MBD for Ni(II)-mediated DNA recognition (44). However, DNA binding in the absence of nickel was not investigated. In the current study, mutating Trp54 to alanine was shown to uncouple nickel and DNA binding, indicating that Trp54 is essential for the allosteric response. Due to the ability of apo- and Ni(II)-W54A to both bind DNA at concentrations similar to apo-WT HpNikR, it seems that Trp54 plays a greater role in allosteric transmission than it does for orienting the DBDs. The inability of apo- and Ni(II)-bound W54L and W54F to form the same DNA-bound complex as WT HpNikR highlights the importance of the tryptophan side chain itself at that position in the MBD/DBD interface. Mutagenesis and DNA-binding studies of the two MBD lysine residues (Lys64 and Lys140) that form a pocket around Trp54 revealed that when the protein is Ni(II)-bound, a cation-π interaction is likely formed between Trp54 and either lysine residue to propagate the allosteric signal to the DBDs. Therefore, the interactions between Trp54 and the lysine residues at the MBD/DBD interface are key for allosteric regulation in response to nickel. In the absence of nickel, this interaction is less important, and it is instead more important to maintain the pocket created by both Lys64 and Lys140. These results indicate that the interactions at the MBD/DBD interface are different for the apo- and Ni(II)-bound proteins, which could contribute to the differences in their DNA-bound signatures observed by ^19^F-NMR that suggest they form somewhat different DNA-bound complexes (35).

A study using molecular dynamics simulations and NMR spectroscopy showed that nickel binding to the MBD modulates the dynamics of the HpNikR inter-domain linkers and unlocks the movement of the DBDs relative to the MBD, leading to the hypothesis that the linkers are important for the allosteric response (50). Allosteric effects through flexible linkers have been well established (59, 86, 87, 88, 89), and the longer length of the linkers in HpNikR compared to its homologs suggested a possible functional significance, making them a likely candidate for allosteric transmission. However, changing the length or flexibility of the inter-domain linkers through the Δ3aa or GGS mutations did not affect DNA binding by Ni(II)-HpNikR. Although the DNA-binding ability of the apo-mutants was impaired, the results indicate that the inter-domain linkers are not involved in propagating the allosteric response. Longer inter-domain linkers likely afford greater flexibility in recognizing more DNA motifs; it is possible that HpNikR has longer linkers than its homologs so that it can effectively bind its loosely conserved recognition sequence in the various promoters of its regulon (37, 38).

Allosteric pathways are typically characterized by long timescale (microseconds to milliseconds) dynamics and are therefore difficult to validate by all-atom molecular dynamics simulations. We chose to perform rigidity-theory based RTA analysis due to its computational efficiency (90) and because it can access motions on millisecond timescales (73, 91). Nevertheless, the current system poses some challenges for computational analysis. First of all, HpNikR is a tetramer, where point mutations are duplicated in each of the four sites. It is also likely that allosteric pathways are distributed asymmetrically across the tetramer (as suggested by our RTA analyses), which is difficult to validate experimentally without separately viewing a response in each protomer. Moreover, RTA predicts allosteric responses assuming a representative structure, but HpNikR undergoes gross conformational changes from *trans* (or possibly open) to *cis* when it binds to DNA, and presumably the DNA would also undergo conformational changes in a 2-site binding model. In ideal circumstances, we would also have high resolution structures of the protein-DNA complex in the *trans* (or open) state where one DBD is bound, in addition to a structure of the apo-protein in complex with DNA. Despite these challenges, the results from the RTA analyses combined with our mutagenesis and DNA-binding studies provided new insights into allosteric transmission in HpNikR.

The RTA analyses on Trp54 and Lys134—the two residues that showed the largest effect on allosteric coupling in the mutagenesis studies—predicted allosteric transmission pathways from either residue to the nickel-binding residues, as well as high transmission to Lys64 and Lys140, supporting the hypothesis based on the experimental data that the residues at the inter-domain interface are all part of the allosteric network linking the nickel sites and the DBDs. Low allosteric transmission to the residues mutated in the inter-domain linkers was predicted, as validated by the lack of an impact on Ni(II)-activated DNA binding by Δ3aa or GGS in the FA assay. In addition, the levels of allosteric transmission vary between monomers, indicating that allostery is asymmetric. Asymmetry in the HpNikR tetramer has previously been observed in a crystal structure of the HpNikR MBD, where subtle differences between the two opposite sides of the MBD were detected based on the distribution of B-factors (43). In addition, the crystal structures of HpNikR show a more asymmetric MBD compared to the symmetric MBD in both EcNikR and PhNikR (42, 45, 49). Therefore, the asymmetry in the HpNikR tetramer likely plays an important role in its allosteric mode of regulation in response to nickel.

A region of the DNA sequence displays a higher level of allosteric transmission in the RTA analysis of Trp54, which corresponds to the half site of the HpNikR pseudo-palindromic recognition sequence containing thymine at position 10. This thymine is known to be essential for tight binding (37, 38). The high degree of transmission to this base, and the DBD that binds it, makes it the likely source of the asymmetric *cis* conformation adopted by Ni(II)-HpNikR in complex with the *ureA* promoter, as observed in our previous ^19^F-NMR study (35), as well as in another study that introduced nuclease activity to HpNikR and detected asymmetric cleavage patterns when bound to this promoter (75). This result also supports the theory first proposed in our ^19^F-NMR study (35) that the asymmetry in the *cis* conformation is a result of the DNA-binding mode for the *ureA* promoter being a two-step process (57). One DBD likely binds the half-site with the thymine first, and then the other DBD is able to bind the other half-site, possibly through a *trans*-intermediate state. This two-step binding model may explain why certain mutants demonstrated lower anisotropy values in the FA experiment and smeared DNA bands in EMSAs. One of the DBDs might bind the half site with the thymine first, but changes to the MBD/DBD interface could affect the ability of the other DBD to bind to the other half site. A two-step binding process also explains why DNA binding fits better to the Hill equation with a Hill coefficient around 2, implying DNA binding is cooperative (35).

The RTA analyses from Lys134 and Trp54 both showed that there is high transmission from the nickel-binding residues through the β-sheets in the MBD to the MBD/DBD interface. A majority of the mutants from the Ni(II)-EcNikR-DNA RTA analysis that did not have a large effect on Ni(II)-activated DNA binding are not found in these β-sheets, except for two residues found on the edges, which is likely why only Lys134 showed a large effect on allosteric coupling. However, it is interesting to note that the apo-mutants typically demonstrated tighter binding to the *ureA* and *ureA*-perf promoters. The tighter apo binding might be because the mutations cause a slight increase in rigidity in the apo-protein or they disrupt interactions that lead to a small shift in the conformational equilibrium toward the more DNA-binding competent states. The FA results using *ureA*-perf showed that nickel and DNA binding are more coupled in some of these mutants compared to the WT protein, whereas only a small decrease in allosteric coupling is observed for all of the mutants with the *ureA* promoter. HpNikR has a large regulon, and it has been shown to bind promoters with different affinities and possibly different conformations (27, 29, 30, 31, 36, 37, 75). The thymine at position 10 is only conserved among promoters that HpNikR binds with high affinity, so it is possible that a different allosteric mechanism, and binding process, is used for a low affinity promoter (38). In a previous study using EMSAs, Ni(II)-K48A was estimated to bind the *ureA* promoter almost an order of magnitude weaker than Ni(II)-WT, whereas only a 2-fold reduction in affinity was observed for the *nixA* promoter (75), which is another HpNikR target known to be bound with an almost identical affinity as the *ureA* promoter (37, 75). The study also showed through the use of several hybrid versions of the *nixA* and *ureA* promoters that any increase in *ureA* sequence content decreased the affinity of Ni(II)-K48A for DNA (75). Those results highlight how the allosteric response can be impacted by the DNA sequence, which supports the differences observed for some of the mutants with the *ureA*-perf *versus* the *ureA* promoter in this study. Taken together, the results of these studies overall indicate the DNA sequence has a significant role in tuning the allosteric response of HpNikR, which likely contributes to the ability of HpNikR to be a global transcriptional regulator in *H. pylori* and bind such a variety of genes. Further dissection of the role of different target promoters on HpNikR allostery provides an avenue for future research.

**Figure 6:**
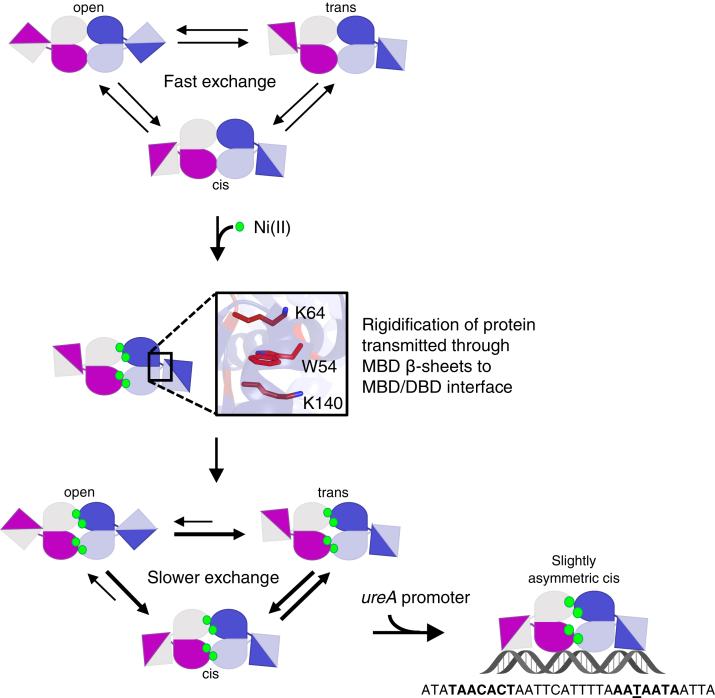
**Schematic of the updated proposed mechanism of Ni(II)-activated DNA binding by HpNikR.** In the absence of nickel and DNA, the protein is in fast exchange between different conformational states due to fast mobility of the DBDs. Nickel binding causes an increase in rigidity that is transmitted through the MBD β-sheets and to the DBDs through interactions between Lys64 or Lys140 and Trp54 at the interface. This slows down the conformational exchange, shifting the conformational equilibrium to the DNA-binding competent *cis*/*trans* states to promote a two-step DNA-binding process involving the crucial thymine (underlined) on one half-site of the *ureA* promoter. The protein is then stabilized in a slightly asymmetric *cis* conformation upon complex formation with the *ureA* promoter. DBD, DNA-binding domain; MBD, metal-binding domain.

Based on this work, we propose a revised model (35) where nickel binding induces long-range allosteric effects, leading to an overall rigidification of the protein that is propagated through the MBD β-sheets to Lys64 or Lys140 at the MBD/DBD interface. These residues form a cation-π interaction with Trp54 to transmit the allosteric signal to the DBDs. This in turn slows down the DBD mobility, shifting the conformational equilibrium to the DNA-binding competent *cis*/*trans* states to promote the two-step binding process (57), wherein one DBD first binds to the crucial thymine on one half-site of the *ureA* promoter. The other DBD is likely then able to bind the second half-site, possibly though a *trans* intermediate state, thus allowing the protein to adopt a slightly asymmetric *cis* conformation upon forming a complex with the promoter (Fig. 6).

For many metalloregulators, allosteric effects upon metal binding are transmitted through changes in H-bonding networks linked to the metal-binding residues (10). In a few metalloregulators, changes in internal dynamics upon metal binding were more recently determined to be important for the allosteric response (10). For example, zinc binding to CzrA from *Staphylococcus aureus* causes a redistribution of fast methyl side-chain dynamics throughout the protein, quenching a network of conditional motions to inhibit DNA binding (60). In addition, for the AdcR metalloregulator from *Streptococcus pneumoniae*, zinc binding was found to enhance internal dynamics in its DBDs to promote interactions with its target DNA sequences (61). In the crystal structures of HpNikR, no clear H-bonding network could be identified through the β-sheets of the MBD. It is possible that HpNikR might be similar to CzrA and AdcR, such that nickel binding could cause an increase in protein rigidity through quenching internal dynamics, particularly throughout the MBD β-sheets. This could provide an explanation for why tighter DNA binding was observed by the apo-mutants selected from the RTA analysis; perhaps substituting those residues for alanine influenced the side-chain dynamics of the apo-protein, leading to tighter DNA binding. Examining the internal dynamics of HpNikR is an avenue of future research, which could help determine how an increase in rigidity is propagated through the MBD β-sheets.

The model proposed here for nickel-activated DNA binding by HpNikR is based on the premise that HpNikR binds nickel in the four square-planar His_3_Cys sites conserved in many NikR homologs (43, 46, 47, 48, 49, 55). However, we do note that there are crystal structures of HpNikR that contain additional coordination sites. These additional sites are thought to be due to differences in purification and crystallization conditions, but their biological relevance is unclear (42, 44, 45). Although the His_3_Cys nickel sites are present in EcNikR (46, 47, 48, 55), there are some differences in the allosteric mechanism of EcNikR compared to HpNikR (10). The allosteric responses of metalloregulators generally fall on a continuum from large conformational changes to subtle changes in dynamics (10). In HpNikR, nickel induces relatively large changes in protein dynamics and conformational equilibria compared to in EcNikR (10, 35). Nickel binding produces short-range effects in EcNikR, inducing a disorder-to-order transition of α-helix 3 and its proceeding loop that contains a Ni(II)-binding residue (47, 52, 92). Ordering of this region is thought to localize EcNikR to DNA through nonspecific electrostatic contacts, allowing the protein to initiate a one-dimensional search along the DNA (48, 93). As it moves along the DNA, the EcNikR DBDs make transient contacts, only adopting the “*cis*” conformation upon finding the recognition sequence (48, 93). RTA analysis on HpNikR shows low allosteric transmission to the α3 helices in the Ni(II)-HpNikR-DNA crystal structure. However, the overall proposed model for both EcNikR and HpNikR seem to be functionally analogous to that of other metalloregulators where DNA binding is activated by metal binding (*i.e.*, the MarR, Fur, and DtxR families) (62). For members of these families, forming a complex with DNA in the absence of its cognate metal is structurally possible but strongly opposed by an unfavorable reduction in the conformational entropy, which only is compensated by the enthalpy of formation of metal ligand coordination bonds with its cognate metal, leading to a favorable Δ*G*_c_ upon DNA binding (10, 13, 62).

Although metalloregulators typically require the cognate metal for DNA binding, HpNikR is unique in that it also responds to changes in pH—consistent with its role as a “master regulator”—so that it can bind DNA with high affinity in the acidic environment *H. pylori* encounters in the human stomach, even in the absence of nickel (28, 30, 74, 94). Both nickel and lower pH cause HpNikR to bind to the *ureA* promoter with similar affinities and locations on the DNA (74), but the mechanism of allosteric regulation by acidity is unknown. Future studies will focus on determining the allosteric response to acidity. Furthering our knowledge on the different modes of allosteric regulation in HpNikR will help advance our understanding of the nickel homeostasis and acid adaptation pathways in *H. pylori* as well as the role of HpNikR in its pathogenicity.

## Experimental procedures

### Vector construction

The pET24bhpnikRG27 vector was generated as previously described (95) and used as the template to prepare the HpNikR mutants. Site-directed mutagenesis was performed according to the QuikChange site-directed mutagenesis protocol (Stratagene) by using primers listed in Table 3, to generate the HpNikR mutants, except K64A/K140A. The K64A/K140A double mutant was generated by introducing the K64A mutation *via* Phusion mutagenesis (Thermo Fisher Scientific), using the forward primer 5′–CTAATGACGAGAGCGCAATCGCCGTG-3′ and reverse primer 5′-GGTTGTCTTCTGCCCAATTG-3′ into a plasmid already containing the K140A mutation. Similarly, the K64M/K140M mutant was prepared by introducing the K64M mutation *via* QuikChange mutagenesis into a plasmid already containing the K140M mutation. Primers were purchased from Integrated DNA technologies and incorporation of each mutation into the plasmid was verified by DNA sequencing (ACGT Corporation and TCAG, The Centre for Applied Genomics, The Hospital for Sick Children).

**Table 3:** Primers for QuikChange site-directed mutagenesis

Mutation	Primer sequence (5′ to 3′)
W54A	GGGTTGTCTTCTGCCGCATTGTCTTCTACTAATTTTTCTCTGATCA
W54L	GGGTTGTCTTCTGCCAAATTGTCTTCTACTAATTTTTCTCTGATC
W54F	ATTAGGGTTGTCTTCTGCAAAATTGTCTTCTACTAATTTTTCTCTGATCATG
K140A	AGGGGGGTTAAATTCGCTAAATTGACTGCGGCGTCTAGCTTT
K64A	CCCTAATGACGAGAGCGCAATCGCCGTGCTTGTGG
K140M	GGGTTAAATTCGCTAAATTGACTATGGCGTCTAGCTTTG
K64M	CCCTAATGACGAGAGCATGATCGCCGTGCTTGTGGT
Δ3aa	TTGGGCAGAAGACAACGAGAGCAAAATCGCCG
GGS	CCACAAGCACGGCGATTTTGCTCTCGCTACCACCGTTGTCTTCTGCCCAATTGTCTTCTA
K31A	TAGACGAATTAGACAACCGCATCATTGCAAACGGCTATTCTTCTCG
N32A	TCGAGAAGAATAGCCGGCTTTAATGATGCGGTTGTCTAATTCGTCT
K48A	GCCCAATTGTCTTCTACTAATGCTTCTCTGATCATGTCGCGCAC
E56A	CGTCATTAGGGTTGTCTGCTGCCCAATTGTCTTCT
E62A	CACGGCGATTTTGCTCGCGTCATTAGGGTTGTC
Q81A	CTGAATGTCTATCATGCGCGCGTTTAATTCCCTTTGGTGGT
T92A	GTGCATAAAACATGCGCCCCGCTGGCATGCTG
I127A	CCTAAGCCCCCCAGCTTCCAATTGCAAGCGTTGGATTTCA
K134A	CCTTAGTCAATTTAGCGAATGCAACCCCCCTAAGCCCCCCA

The second primer for each is the reverse complement.

### Protein expression and purification

All plasmids were transformed into and expressed from BL21(DE3) *E. coli* cells as previously described (35, 95). Briefly, cells were grown at 37 °C in 3l of LB media containing 50 μg/ml kanamycin until an OD_600_ of 0.6 to 0.9 was reached. Protein expression was then induced with 0.3 mM IPTG for 4 h at 37 °C. After harvesting, cells were resuspended in protein buffer (20 mM Tris, pH 7.6 and 100 mM NaCl) and lysed by sonication. The lysate was passed through a 0.45 μm syringe filter and then loaded onto a DEAE Sepharose anion-exchange column (GE Healthcare) equilibrated with 20 mM Tris, pH 7.6 and then eluted with a linear NaCl gradient increasing up to 1 M. 12% SDS-PAGE was used to identify fractions containing the desired protein, which were pooled and dialyzed overnight against 20 mM Tris, pH 7.6, 1 mM DTT, and 10 mM EDTA at 4 °C. The dialyzed protein was then loaded onto MonoQ (GE Healthcare) anion exchange chromatography column with the same pre-equilibration and elution conditions used as the DEAE column. Fractions were again analyzed by 12.5% SDS-PAGE, and those containing the desired protein were collected and stored at 4 °C.

Protein concentrations were determined in protein buffer by measuring the electronic absorption at 280 nm and using an extinction coefficient of 8480 M^−1^ cm^−1^ or 2980 M^−1^ cm^−1^ for W54F HpNikR (96). For W54A and W54L HpNikR, an extinction coefficient of 5595 M^−1^ cm^−1^ was determined by amino acid analysis (SPARC BioCentre, The Hospital for Sick Children). The molecular mass of each protein was confirmed by electrospray ionization mass spectrometry (AIMS Laboratory, University of Toronto). The oxidation state of the protein was determined using a 5,5′-dithiobis-(2-nitrobenzoic acid) (DTNB) assay. For the DTNB assay, protein samples and 7 to 56 μM β-mercaptoethanol standards were prepared in 6 M GuHCl, 1 mM EDTA (pH 8.0), and 400 μM DTNB and the absorbance was measured at 412 nm. The protein was used if it was over 90% reduced.

### Nickel titration

Electronic absorption spectra were collected at 25 °C on an Agilent 8452 spectrophotometer. Increasing amounts of NiSO_4_ were titrated into a fixed concentration of protein every 10 min at 25 °C and the absorbance was measured at 302 nm. The difference absorbance spectrum was generated by subtracting the spectrum of the apo-protein from that of the Ni(II)-bound protein.

### CD spectroscopy

CD spectra were recorded on an Olis DSM 1000 spectrometer at 20 °C. Samples of 20 μM protein were prepared and dialyzed overnight at 4 °C against 100 mM potassium phosphate buffer, pH 7.6. The CD spectra were acquired across 190 to 260 nm at 1 nm intervals with an integration time of 2 s. A total of five replicate spectra were collected and averaged to obtain the final spectrum for each sample. The measured ellipticity (θ, mdeg) was converted to mean residue ellipticity [θ] (deg cm^2^ dmol^−1^) using equation (Equation 1):

(1) $$\left[\theta \right]=\frac{\theta \times 100}{nlc}\text{,}$$

where *n* is the number of residues, *l* is the path length (cm), and *c* is the protein concentration (mM).

### Fluorescence anisotropy (FA) assay and Δ*G*_c_ calculations

The 32 bp fluorescein [F] labeled and unlabeled oligonucleotides containing the HpNikR recognition sequence from the *ureA* promoter and the modified *ureA*-perf promoter were purchased from Integrated DNA Technologies: *ureA*, 5′-ATATAACACTAATT[F]CATTTTAAATAATAATTA-3′ and 5′-TAATTATTATTTAAAATGAATTAGTGTTATAT-3′; *ureA*–perf, 5′-ATATATTATTAATT[F]CATTTTAAATAATAATTA-3′ and 5′-TAATTATTATT- TAAAATGAATTAATAATATAT-3′. The fluorescein molecule [F] was conjugated to a central thymine located in the spacer between the two half-sites of the HpNikR recognition sequence, where it is unlikely to interfere with HpNikR binding (34, 35). The oligonucleotides were annealed and quantified as described previously (35).

The nickel-bound proteins used for these experiments were prepared by incubating the protein with 1.2 equivalents of NiSO_4_ per protein monomer in protein buffer supplemented with 3 mM MgSO_4_ for 1.5 h at room temperature. Increasing concentrations of apo- or nickel-bound protein were incubated with the fluorescently labeled *ureA* or *ureA*-perf promoter on a black Nunc 384-well plate for a final concentration of 5 nM oligonucleotide. Measurements were taken on a ClarioSTAR Plus plate reader with an excitation wavelength of 482 nm and an emission wavelength of 540 nm. The data were analyzed by converting the anisotropy, *r*, to fraction bound *F*_bound_ (the fraction of protein bound to DNA at a given DNA concentration), using equation (Equation 2) (34, 97):

(2) $$F_{\text{bound}}=\frac{r-r_{\text{free}}}{\left(r_{\text{bound}}-r\right)Q+\left(r-r_{\text{free}}\right)}\text{,}$$

where *r*_free_ is the anisotropy of fluorescein-labeled oligonucleotide probe, *r*_bound_ is the anisotropy of the DNA-protein complex at saturation, and *Q* is the quantum yield ratio of the fluorescent intensities of the bound to free form, calculated from the change in fluorescence intensity (*Q* = *I*_bound_/*I*_free_). *F*_bound_ was plotted against the protein concentration, always treating the protein as a tetramer (39, 74, 98). The *K*_d_ value was determined by fitting the data to the Hill Equation (Equation 3):

(3) $$F_{\text{bound}}=\frac{{\left[P\right]}^{n}}{K_{d}^{n}+{\left[P\right]}^{n}}\text{,}$$

where *P* is the concentration of protein tetramer, *K*_d_ is the protein concentration needed for 50% binding, and *n* is the Hill Coefficient. The allosteric coupling free energy (Δ*G*_c_) was then calculated from Equation 4:

(4) $$\Delta G_{c}=-RT\;\ln\left(K_{d,\text{apo}}/{K_{d}}_{,\text{Ni}\left(\text{II}\right)-\text{bound}}\right)\text{,}$$

with the error in Δ*G*_c_ determined from the square root of the sum of the squares of the SD of the mean value of *K*_d, Ni(II)-bound_ and *K*_d, apo_ obtained from three experiments (62, 99).

### EMSA

The 32 bp *ureA* promoter was synthesized and HPLC-purified by IDT as a duplex, 5′-ATATAACACTAATTCATTTTAAATAATAATTA-3′. The DNA probes were labeled at both ends with γ-^32^P-ATP (PerkinElmer) using T4 polynucleotide kinase for 2 h at 37 °C. Unincorporated nucleotides were removed with a G-25 microspin column (GE Healthcare) and the amount of labeling incorporated was determined using a Packard TriCarb 2900TR Liquid Scintillation Counter.

Radiolabeled DNA was incubated for 30 min with increasing concentrations of nickel-bound protein at room temperature in binding buffer (20 mM Tris, pH 7.5, 100 mM KCl, 3 mM MgCl_2_, 0.1% octylphenoxypolyethoxyethanol (IGEPAL), 5% glycerol, and 0.1 mg/ml sonicated herring sperm DNA (Promega)). The reactions were resolved on 10% native Tris-Borate (300 mM boric acid and 75 mM Tris, pH 7.5) polyacrylamide gels containing 800 μM NiSO_4_ for 2 h at 350 V and 4 °C after pre-running the gel for 1 h in TB running buffer (300 mM boric acid and 75 mM Tris–HCl, pH 7.5, with 800 μM NiSO_4_). The gel was vacuum-dried and exposed overnight to a phosphor screen, scanned with a Pharos Fx Plus Molecular Imager (BioRad), and analyzed with Quantity One software.

### Rigidity and allostery calculations

An extension of the method FIRST (69, 71) was utilized to perform rigidity theory-based structural analysis. Starting with a crystal structure, FIRST was used to create a geometric molecular framework, whose underlying network (graph) consists of atoms (nodes) and edges (*i.e*., constraints representing covalent bonds, hydrogen bonds, electrostatic interactions, and hydrophobic contacts). Every potential hydrogen bond is assigned an energy strength in kcal/mol, and a hydrogen bond cutoff energy value is selected so that all bonds weaker than this cutoff are removed from the network. Using a fast-exact combinatorial pebble game algorithm (68, 69, 70), FIRST rapidly decomposes a protein structure into flexible and rigid clusters while incrementally removing current weakest hydrogen bond. Allostery analysis on Ni(II)- and DNA-bound EcNikR (PDB: 2HZV) (48) and HpNikR (PDB:6MRJ) (56) crystal structures was carried out by applying RTA analysis (67). The RTA method (67, 100) identifies allosteric networks within structures of proteins and protein complexes. We applied the RTA algorithm by rigidifying DNA in the Ni(II)- and DNA-bound EcNikR structure (PDB: 2HZV) (48) and observing changes in degrees of freedom and allosteric response in the rest of the complex. In the Ni(II)-HpNikR-DNA structure (56), RTA was also applied by measuring the allosteric influence of Trp54 and Lys134.

## Data availability

Data related to the computational analysis can be obtained upon reasonable request from corresponding author Adnan Sljoka (adnan.sljoka@riken.jp). The rest of the data are contained within the article and the supporting information.

## Dedication

This paper is dedicated to the memory of Dr Deborah B. Zamble, an incredible scientist, mentor, and colleague.

## Supporting information

This article contains supporting information (32, 74, 101, 102, 103, 104, 105).

## Conflict of interest

The authors declare that they have no conflicts of interest with the contents of this article.
