# Supplementary material for: Mechanistic insights into the nickel-dependent allosteric response of the Helicobacter pylori NikR transcription factor
Source: J Biol Chem. 2022 Dec 9;299(1):102785. doi: 10.1016/j.jbc.2022.102785 (PMC9860126; doi:10.1016/j.jbc.2022.102785)
Supplement: Supplemental Figures S1–S12 and Tables S1, S2 [file mmc1.pdf]

Supporting Information for:

Mechanistic insights into the nickel-dependent allosteric response of the *Helicobacter pylori* NikR transcription factor

Karina A. Baksh[1], Jerry Augustine[2], Adnan Sljoka[3], R. Scott Prosser[1,2], and Deborah B. Zamble[1,2]

[1]Department of Biochemistry, University of Toronto, Toronto, Ontario, Canada M5S 1A8
[2]Department of Chemistry, University of Toronto, Toronto, Ontario, Canada M5S 3H6
[3]RIKEN Center for Advanced Intelligence Project, RIKEN, 1-4-1 Nihombashi, Chuo-ku, Tokyo, 103-0027 Japan

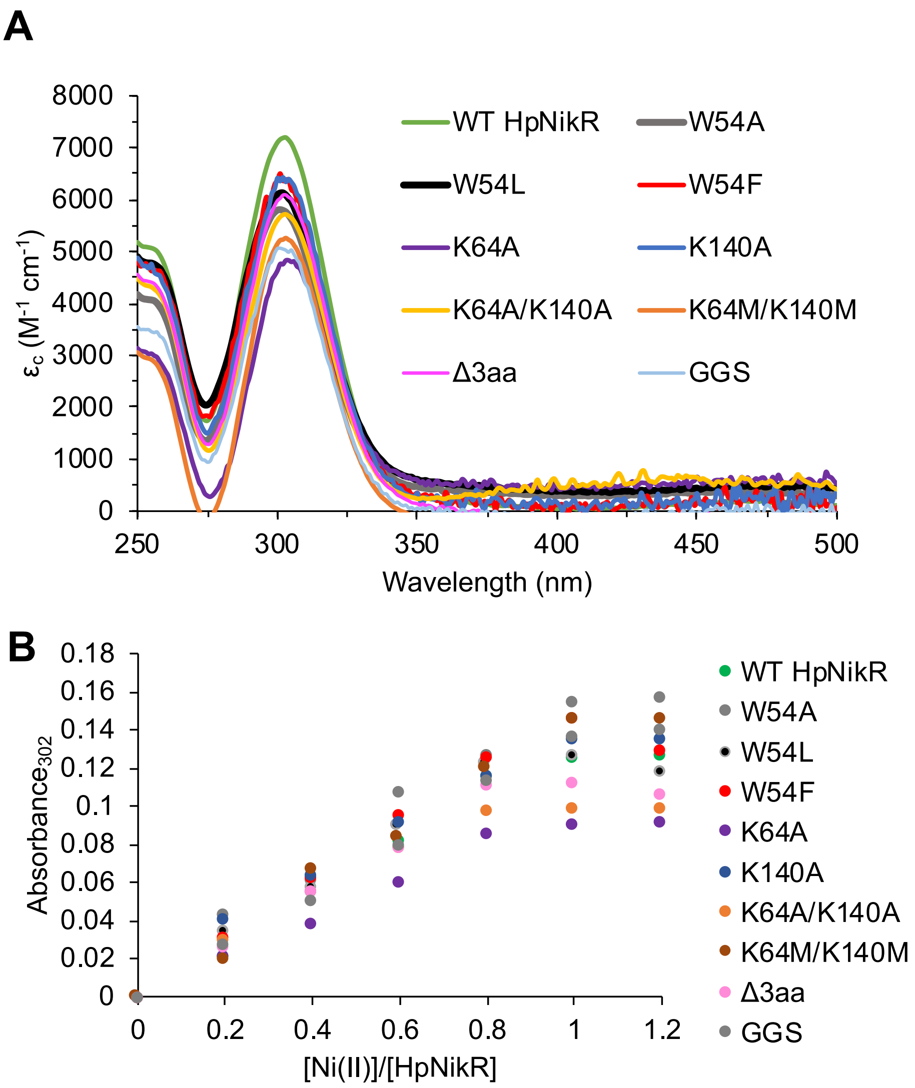

Figure S1. Nickel binding to WT HpNikR and mutants. The change in electronic absorption was monitored upon titrating nickel into a fixed concentration of protein. **(A)** The difference electronic absorption spectrum of nickel bound to WT or mutant HpNikR – generated by subtracting the spectrum of the apo-protein from the Ni(II)-saturated protein – shows an intense peak at 302 nm with extinction coefficients (ε_c) between 5000-7000 M^{-1} cm^{-1}, indicating a ligand-to-metal charge transfer from cysteine to nickel. These spectra are characteristic for HpNikR and four-coordinate nickel bound in a square planar or distorted square planar geometry (1-3). Therefore, nickel coordination is not affected in any mutant. **(B)** Titration of WT HpNikR and mutants with increasing amounts of nickel produces a linear increase in absorbance at 302 nm until saturation near 1 equivalent of nickel, indicating a stoichiometry of one nickel ion per monomer. Therefore, stoichiometric nickel binding is also retained in every mutant.

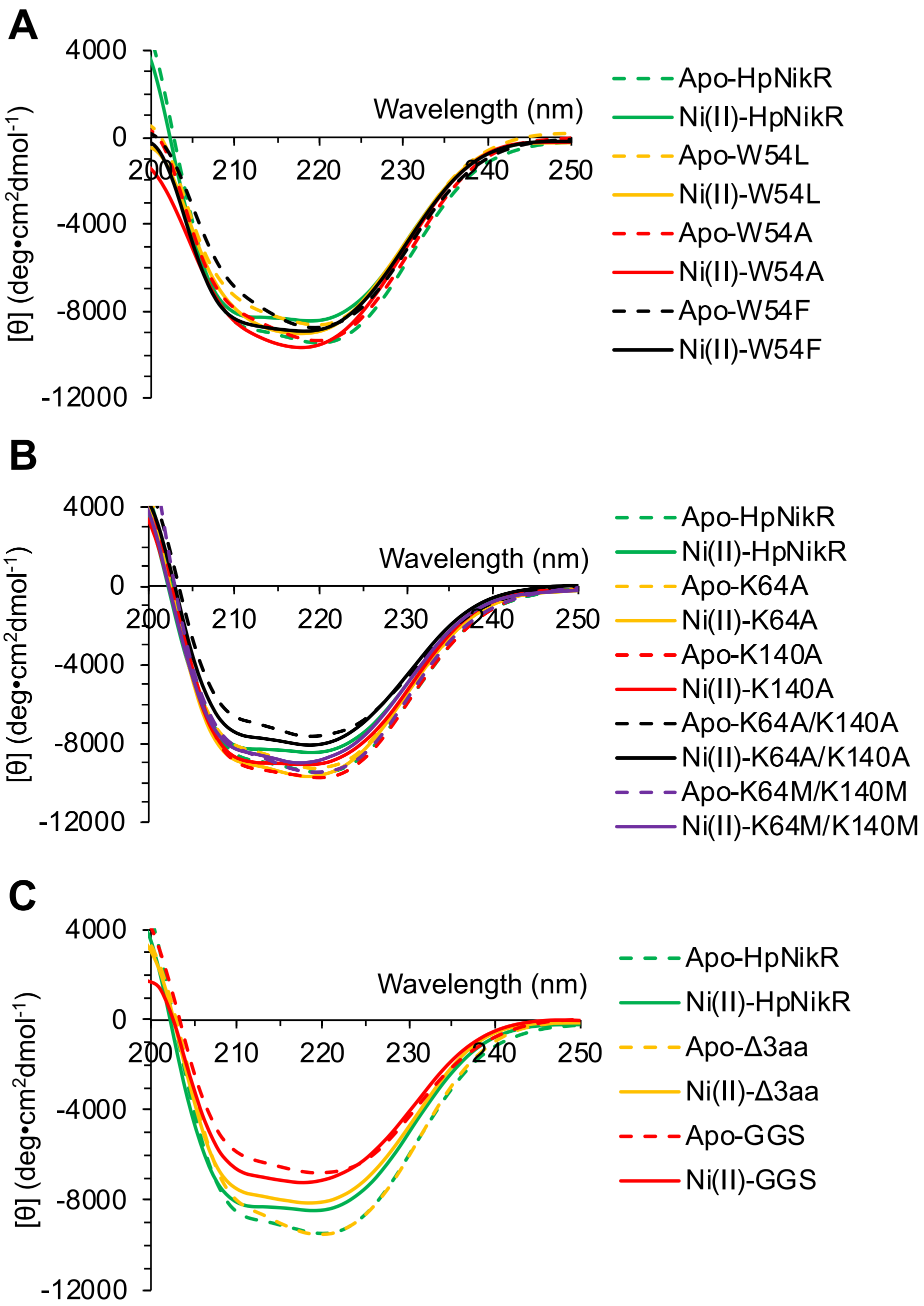

Figure S2. Secondary structure of WT HpNikR and mutants. The circular dichroism spectra of apo and Ni(II)-bound WT HpNikR, **(A)** tryptophan mutants, **(B)** lysine mutants, and **(C)** inter-domain linker mutants all indicate a mixture of α-helices and β-sheets. Therefore, the secondary structure is not disrupted in the mutants. The spectra of WT HpNikR match previously reported spectra (4); in order to compare the spectra of the mutants to the WT protein, the amounts of secondary structural elements were determined using BeStSel, as shown in Table S1.

Table S1. Comparison of secondary structure determinations from circular dichroism spectra shown in Figure S3 using BeStSel (5-7) indicates that the mutations do not disrupt secondary structure.

Protein	% α-helix	% β-sheets	% turns	% unstructured
Apo-WT HpNikR	14	30	13	43
Ni(II)-WT HpNikR	15	31	13	42
Apo-W54A	12	29	15	45
Ni(II)-W54A	11	34	12	43
Apo-W54F	9	31	14	46
Ni(II)-W54F	13	31	15	42
Apo-W54L	13	27	15	45
Ni(II)-W54L	10	34	13	42
Apo-K64A	16	25	13	46
Ni(II)-K64A	17	27	13	44
Apo-K140A	16	27	13	44
Ni(II)-K140A	16	31	13	41
Apo-K64A/K140A	12	30	15	44
Ni(II)-K64A/K140A	15	30	14	42
Apo-K64M/K140M	22	13	10	55
Ni(II)-K64M/K140M	16	27	13	45
Apo-Δ3aa	17	24	14	45
Ni(II)-Δ3aa	15	31	14	40
Apo-GGS	12	26	15	47
Ni(II)-GGS	12	33	15	40

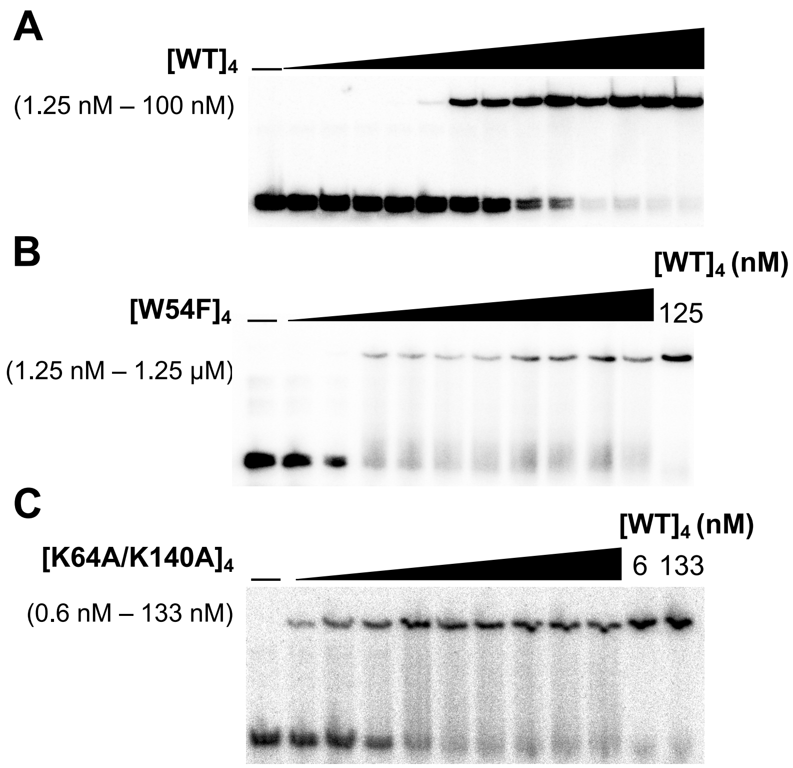

Figure S3. Electrophoretic mobility shift assays showing DNA-binding of WT and mutant HpNikR proteins to the *ureA* promoter. Increasing concentrations of Ni(II)-bound **(A)** WT HpNikR, **(B)** W54F, and **(C)** K64A/K140A were incubated with the 32 bp *ureA* promoter and analyzed on 10% native polyacrylamide gels with excess nickel in the gel and running buffer. Gels shown for W54F and K64A/K140A include WT HpNikR as controls in the last lane and last two lanes, respectively, to compare the shifts of the bands.

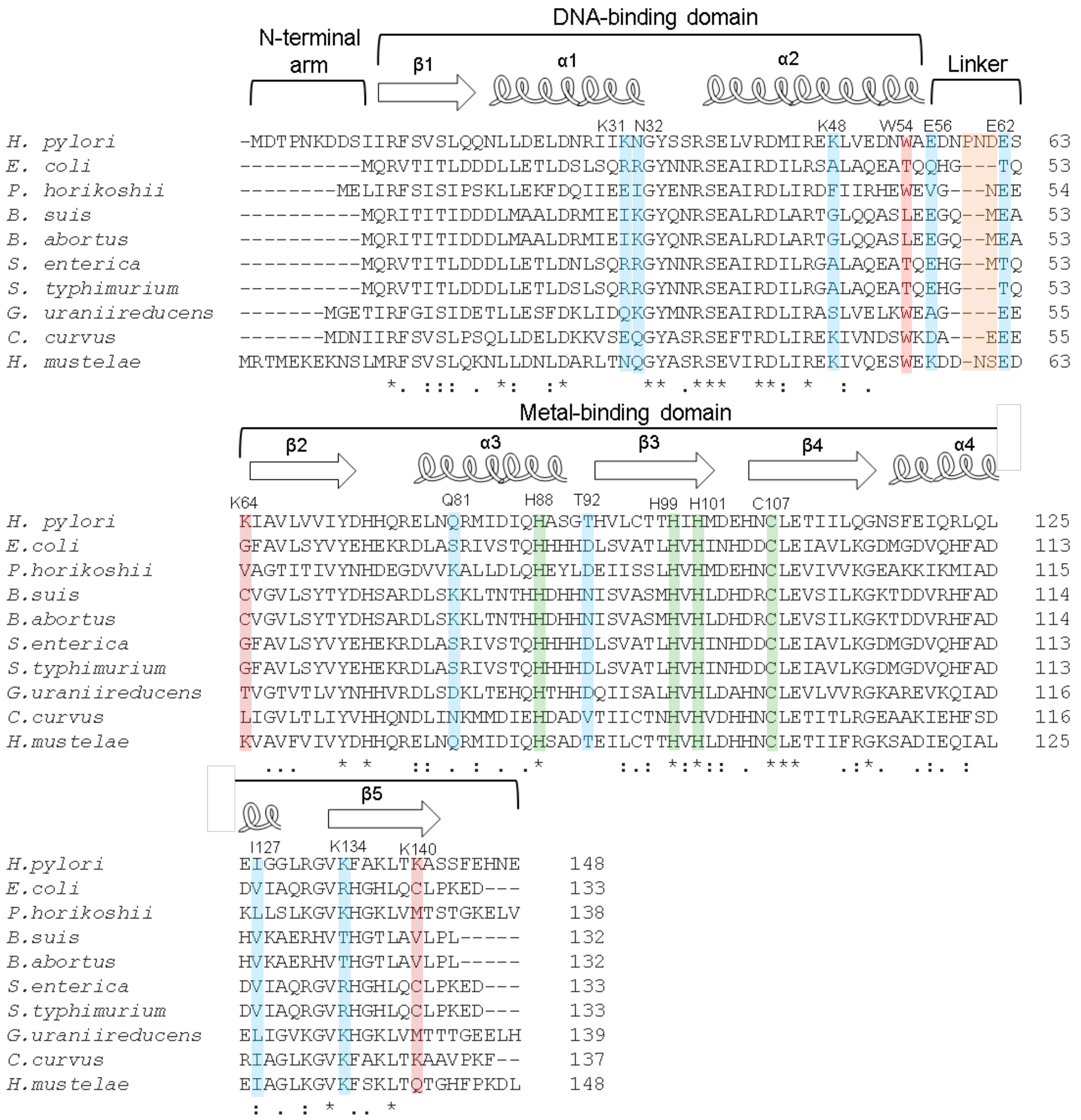

Figure S4. Sequence alignment of NikR homologs and the corresponding HpNikR secondary structure. Asterisks, double dots, and single dots indicate complete conservation, conserved substitutions, and semi-conserved substitutions, respectively. Residues chosen for mutations at the MBD/DBD interface are shaded in red, inter-domain linker mutations are in orange, and mutations based on the RTA analysis are in blue. Residues involved in nickel binding are shaded in green. Protein sequences were collected from the NCBI protein databank and aligned using Clustal Omega.

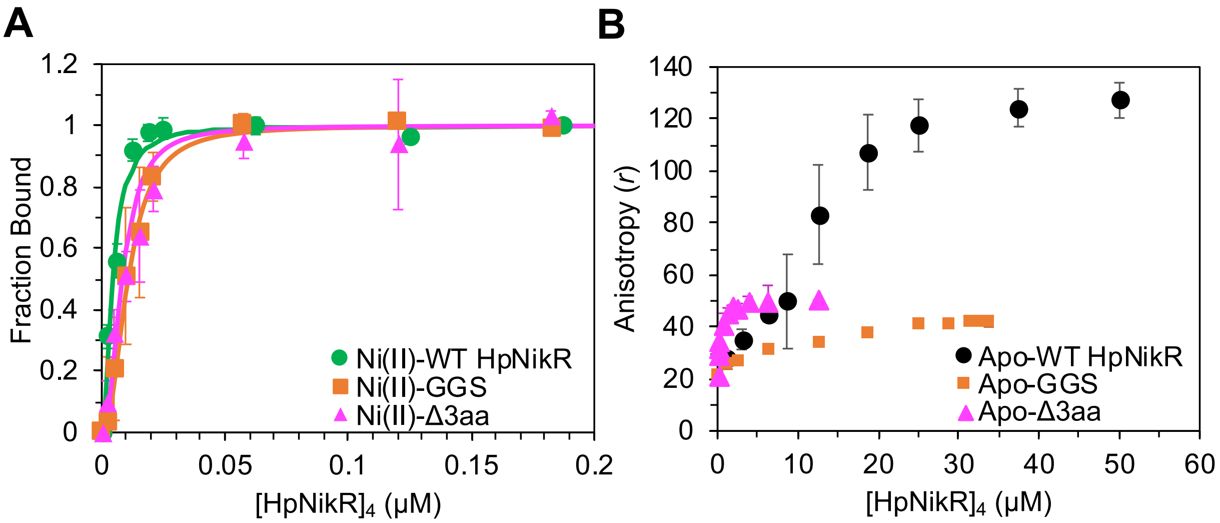

Figure S5. Fluorescence anisotropy-monitored DNA binding of WT HpNikR and inter-domain linker mutants with the *ureA* promoter. **(A)** Ni(II)-GGS and Ni(II)-Δ3aa bind the *ureA* promoter with similar affinities as Ni(II)-WT HpNikR. The data from each replicate were fit to the Hill equation, and the calculated DNA-binding affinities are shown in Table 1. **(B)** Change in anisotropy, *r*, of all of the apo-linker mutants is lower than that of apo-WT HpNikR. Experiments were performed with 5 nM of the *ureA* promoter in 3 mM $MgSO_4$, 20 mM Tris, 100 mM NaCl, pH 7.6. The data points represent the average derived from the preparation of three samples at each protein concentration, and the error bars represent ± one standard deviation.

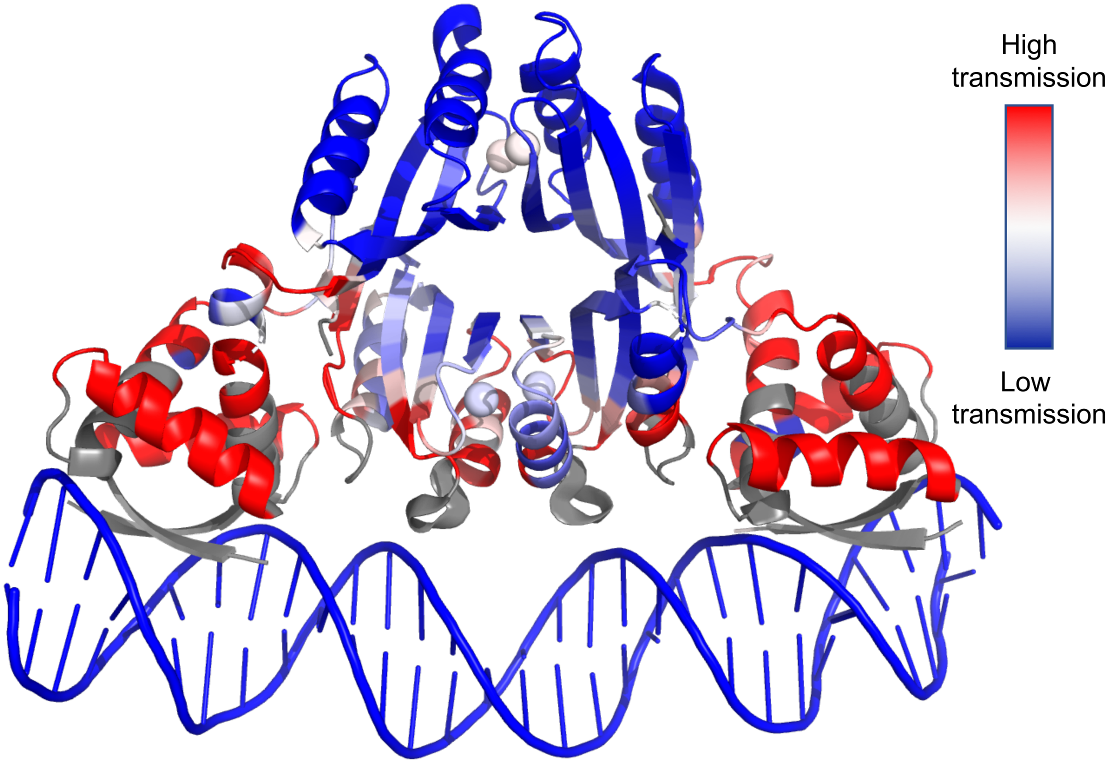

Figure S6. Crystal structure of Ni(II)-EcNikR-DNA (PDB:2HZV) showing allosteric transmission pathways predicted by RTA analysis. Regions are coloured based on the magnitude of allosteric response from the DNA to the rest of the protein. Grey indicates regions that are in close contact with the DNA sequence, which are not suitable for mutagenesis studies.

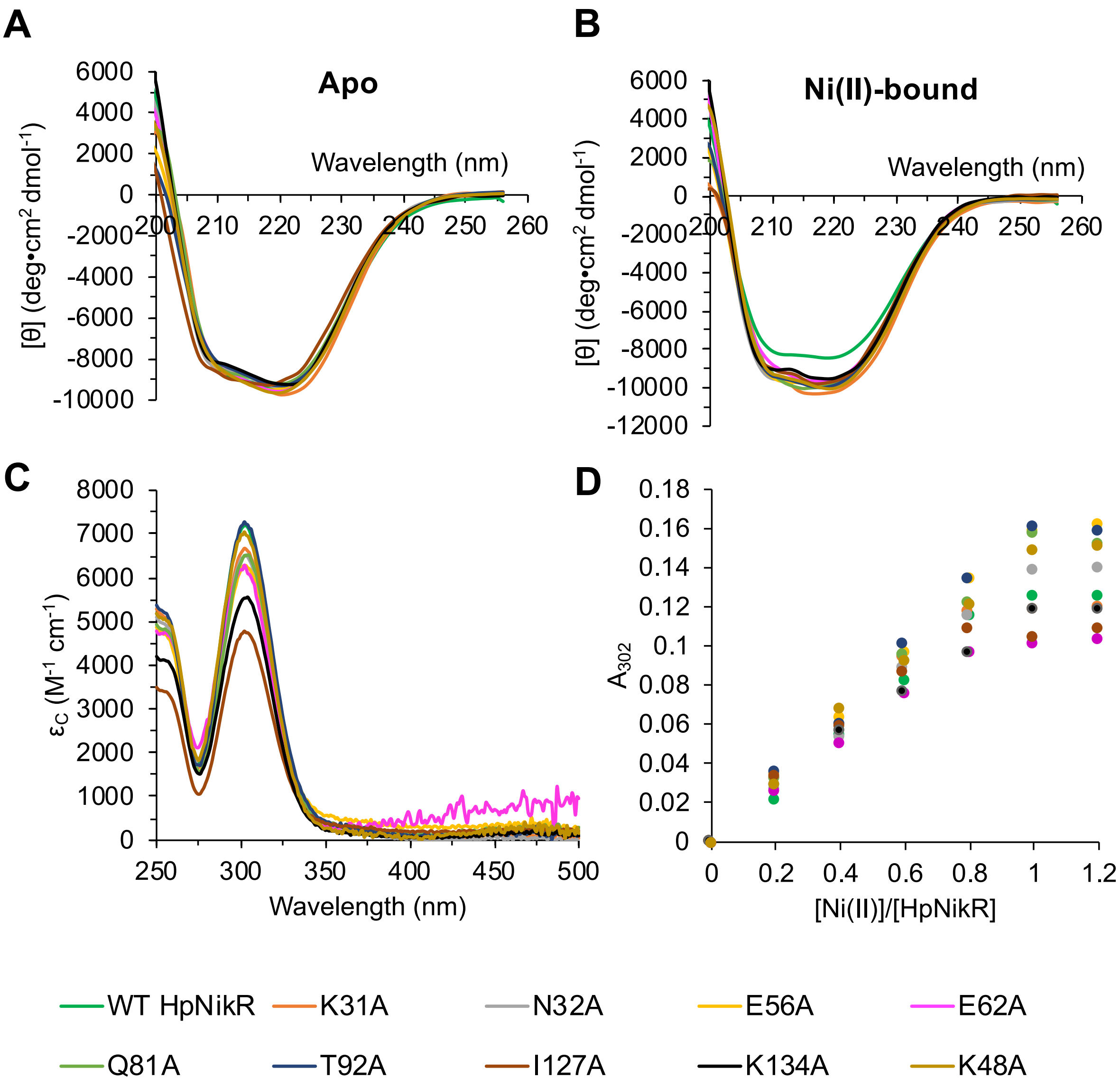

Figure S7. Comparing the nickel-binding activity and secondary structure of WT HpNikR and mutants chosen from RTA analysis. Circular dichroism spectra of **(A)** apo- and **(B)** Ni(II)-bound WT HpNikR and mutants indicate a mixture of α-helices and β-sheets. The spectra of WT HpNikR match previously reported spectra (4); in order to compare the spectra of the mutants to the WT protein, the amounts of secondary structural elements were determined using BeStSel, as shown in Table S2. The secondary structure is not disrupted in any mutant. **(C)** Difference electronic absorption spectra generated by subtracting the spectrum of the apo-protein from the Ni(II)-saturated protein to examine nickel-binding activity shows an intense peak at 302 nm with extinction coefficients (ε_c) between 5000-7000 M^{-1} cm^{-1}, indicating a ligand-to-metal charge transfer from cysteine to nickel. These spectra are characteristic for HpNikR and four-coordinate nickel bound in a square planar or distorted square planar geometry (1-3). Therefore, nickel coordination is not affected in any mutant. **(D)** Titration of WT HpNikR and mutants with increasing amounts of nickel produces a linear increase in absorbance at 302 nm until saturation near 1 equivalent of nickel, indicating a stoichiometry of one nickel ion per monomer. Therefore, stoichiometric nickel binding is also retained in every mutant.

Table S2. Comparison of secondary structure determinations from circular dichroism spectra shown in Figure S7A,B using BeStSel (5-7) indicates that the mutations do not disrupt secondary structure.

Protein	% α-helix	% β-sheets	% turns	% unstructured
Apo-WT HpNikR	14	30	13	43
Ni(II)-WT HpNikR	15	31	13	42
Apo-K31A	16	37	14	33
Ni(II)-K31A	15	29	14	42
Apo-N32A	23	17	13	48
Ni(II)-N32A	21	22	12	45
Apo-K48A	16	23	15	45
Ni(II)-K48A	18	28	13	40
Apo-E56A	17	23	14	46
Ni(II)-E56A	17	29	13	41
Apo-E62A	15	25	14	46
Ni(II)-E62A	23	17	11	49
Apo-Q81A	16	25	15	42
Ni(II)-Q81A	16	31	13	40
Apo-T92A	15	23	15	46
Ni(II)-T92A	17	30	13	41
Apo-I127A	14	25	14	47
Ni(II)-I127A	18	24	15	43
Apo-K134A	18	22	14	45
Ni(II)-K134A	23	19	12	45

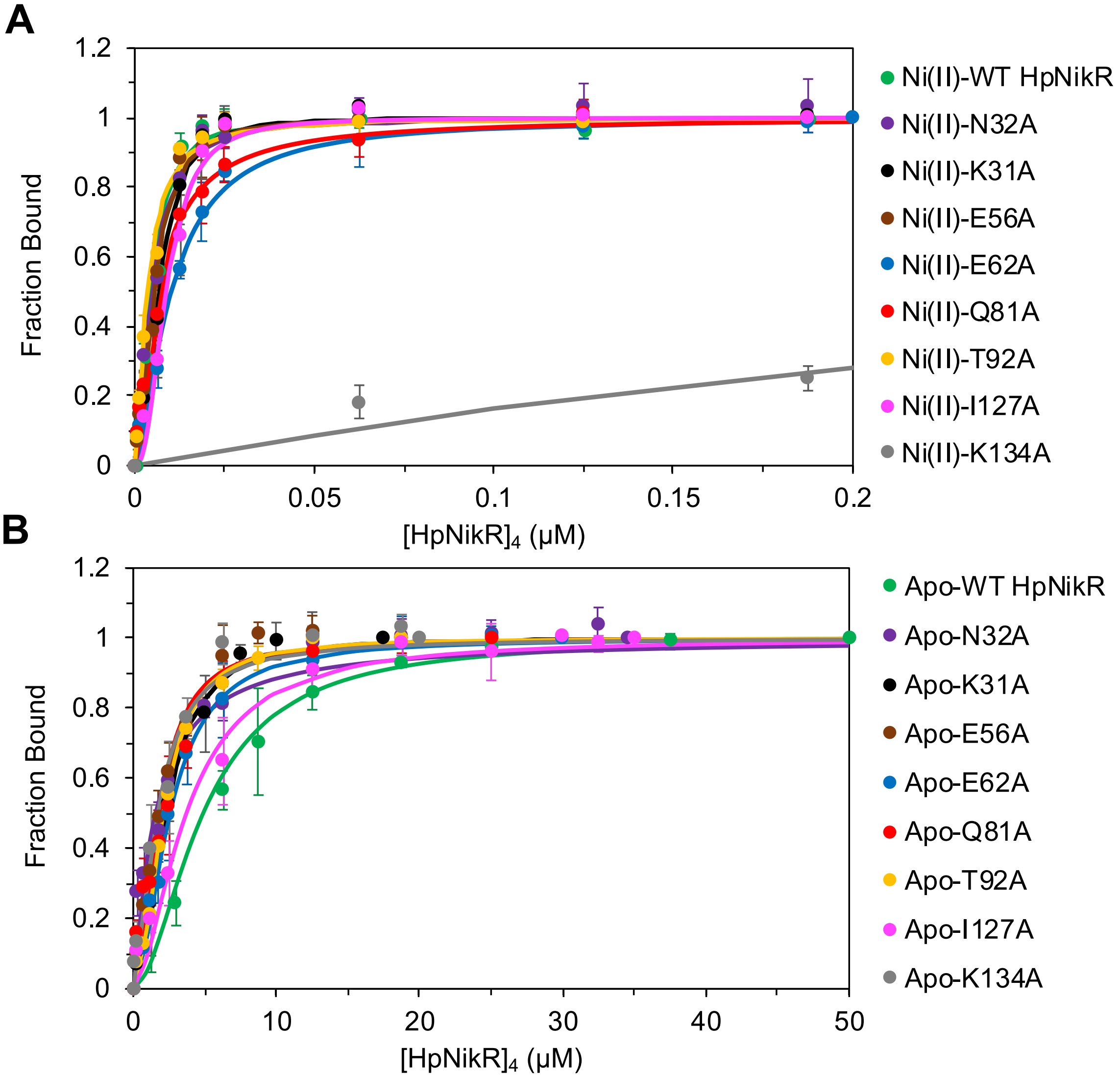

Figure S8. Fluorescence anisotropy-monitored DNA binding of **(A)** Ni(II)-bound and **(B)** apo-WT HpNikR and mutant proteins based on RTA analysis with the *ureA* promoter. Experiments and data processing were performed as described for Figure S5. The calculated DNA-binding affinities are shown in Table 2.

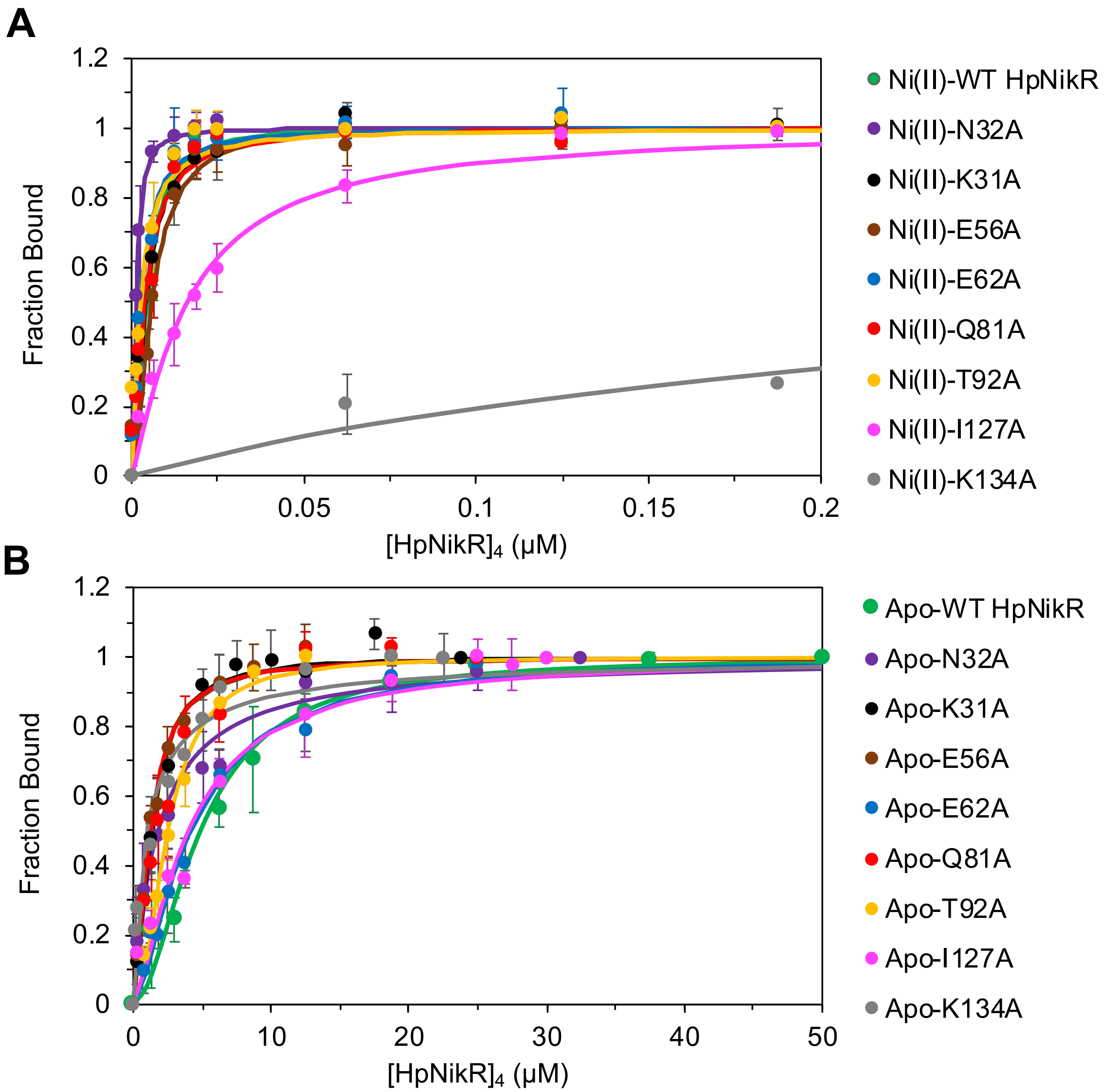

Figure S9. Fluorescence anisotropy-monitored DNA binding of **(A)** Ni(II)-bound and **(B)** apo-WT HpNikR and mutant proteins based on RTA analysis with the *ureA*-perf promoter. Experiments were performed with 5 nM of the promoter in the same conditions as described in Figure S5. Data processing was also performed as described in Figure S5, and the calculated DNA-binding affinities are shown in Table 2.

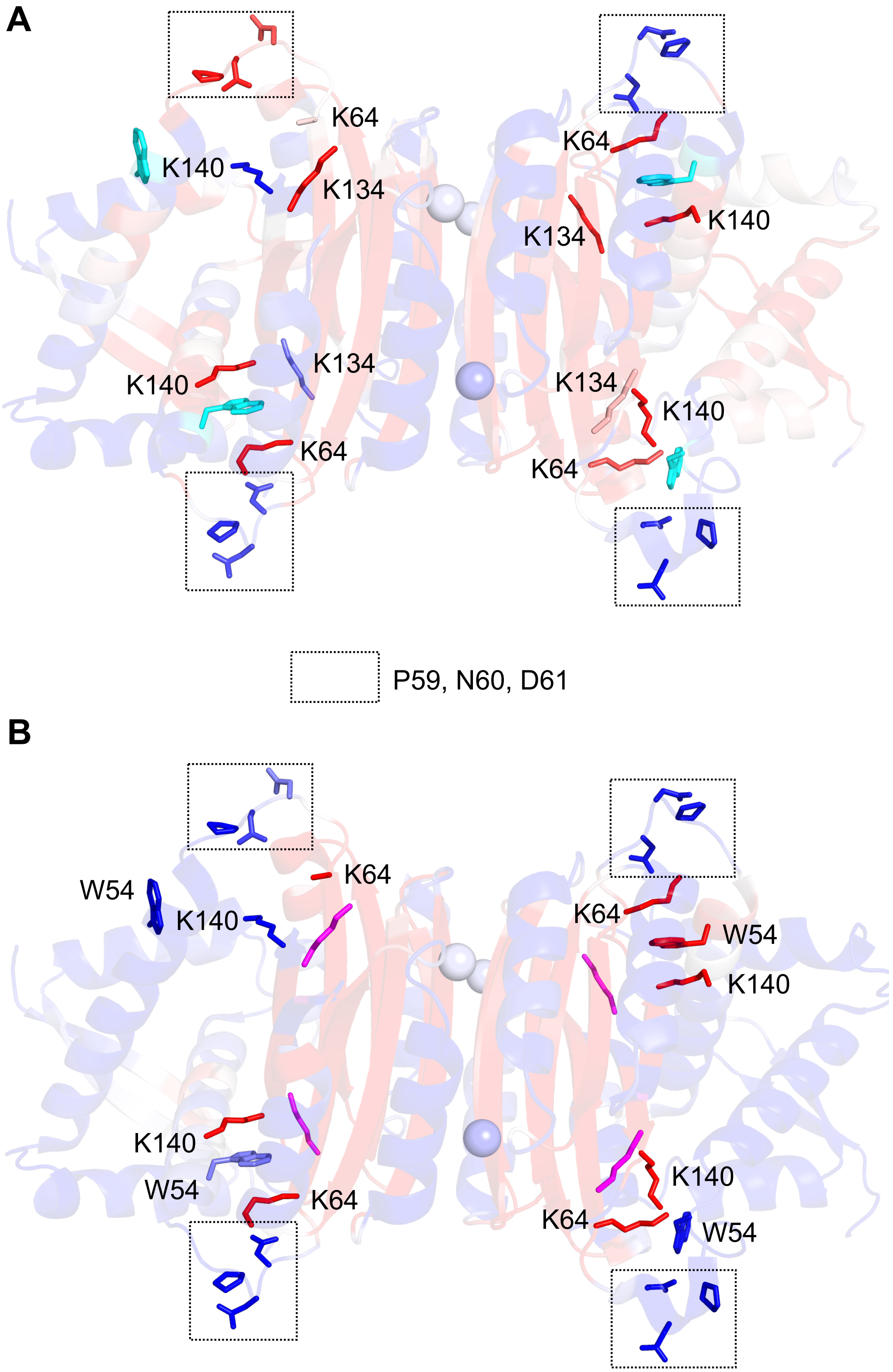

Figure S10. Crystal structure of Ni(II)-HpNikR-DNA (PDB: 6MRJ) showing allosteric transmission pathways predicted by rigidity theory analysis from rigidifying **(A)** Trp54 (cyan) and **(B)** Lys134 (pink), as also shown in Figure 5. Sticks correspond to residues from the mutagenesis studies at the inter-domain interface and inter-domain linkers, and are coloured representing the magnitude of allosteric transmission from either (A) Trp54 or (B) Lys134.

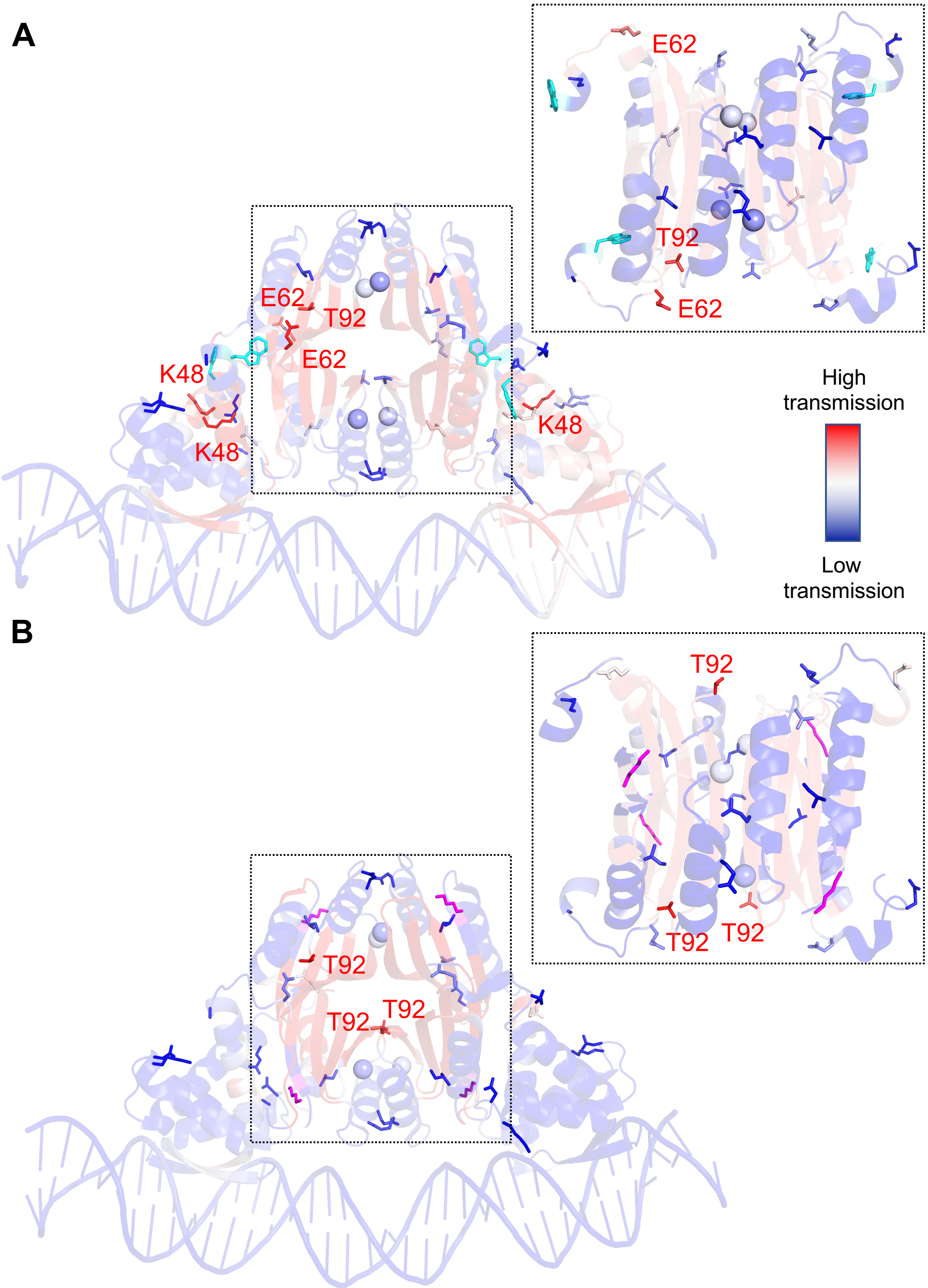

Figure S11. Crystal structure of Ni(II)-HpNikR-DNA (PDB: 6MRJ) showing allosteric transmission pathways predicted by rigidity theory analysis from rigidifying **(A)** Trp54 (cyan) and **(B)** Lys134 (pink), as also shown in Figure 5. Sticks correspond to residues mutated based on RTA analysis and are coloured representing the magnitude of allosteric transmission from either (A) Trp54 or (B) Lys134. Inset shows a rotated view of just the MBD, highlighting that the residues displaying higher allosteric transmission are found on the edges of the β-sheets.

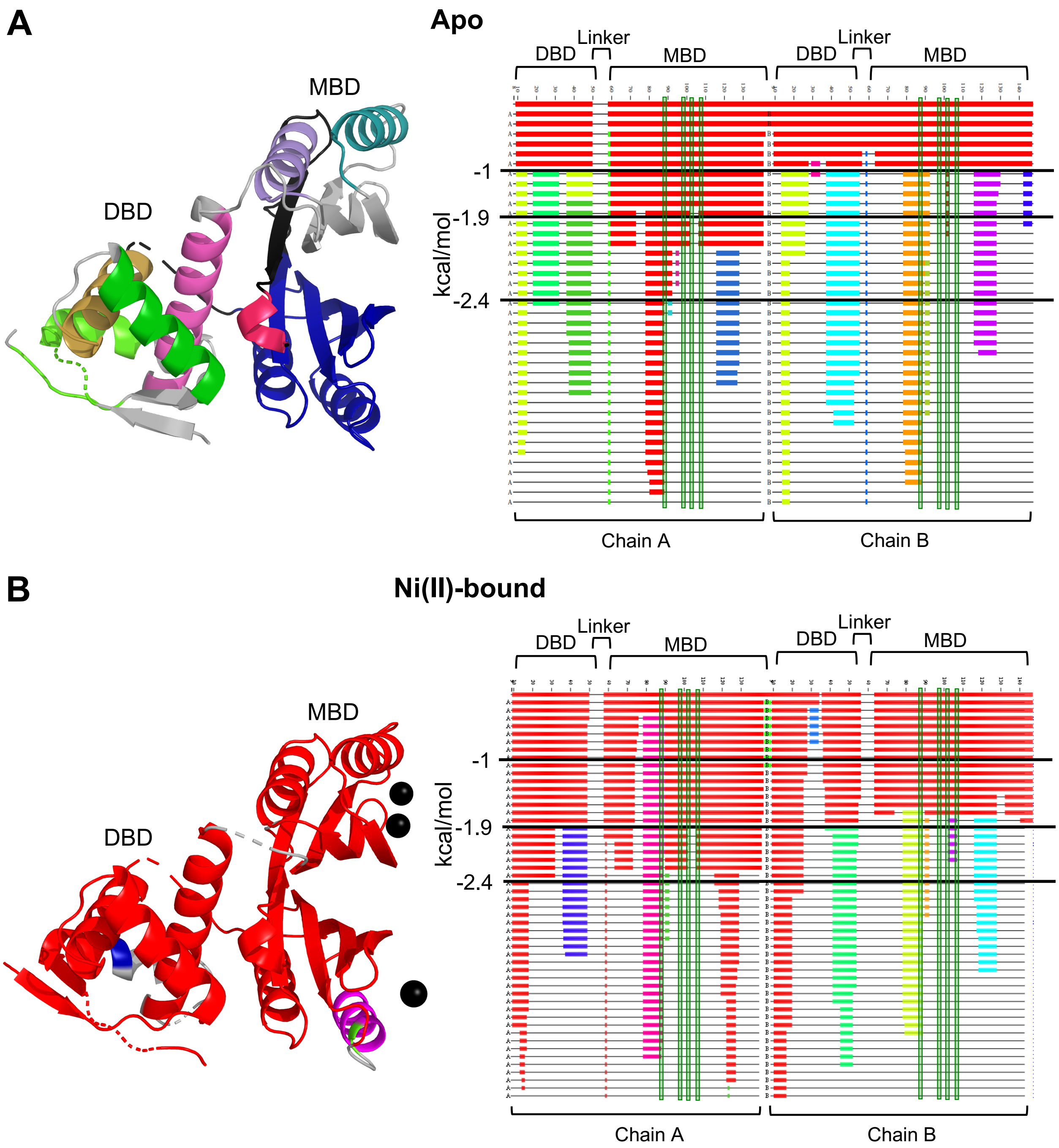

Figure S12. Computational rigidity predictions of crystal structures of **(A)** apo (PDB:2CA9) and **(B)** Ni(II)-bound (PDB:2CAD) HpNikR. (Left) Structures are coloured by rigidity at an energy cutoff of around -1.2 kcal/mol. Red is the largest rigid cluster, and grey regions are flexible parts of the protein. (Right) Hydrogen bond dilution plot. Energy cutoff lines are highlighted in black at -1, -1.9 and -2.4 kcal/mol. Vertical green boxes indicate nickel sites. In (A), rigid cluster decompositions show that after an energy cutoff of -1 kcal/mol, HpNikR is composed of several rigid clusters (indicated by colours) and flexible connections, indicating a loss of overall rigidity. In (B) HpNikR is mostly rigid (as shown by red blocks on dilution plot).

References

1. Chen, X., Chu, M., and Giedroc, D. P. (2000) Spectroscopic characterization of Co(II)-, Ni(II)-, and Cd(II)-substituted wild-type and non-native retroviral-type zinc finger peptides. *J. Biol. Inorg. Chem.* **5**, 93-101
2. Lever, A. B. P. (1985) *Inorganic Electronic Spectroscopy, Studies in Physical and Theoretical Chemistry*, 2 ed., Elsevier Science, New York
3. Abraham, L. O., Li, Y., and Zamble, D. B. (2006) The metal- and DNA-binding activities of *Helicobacter pylori* NikR. *J. Inorg. Biochem.* **100**, 1005-1014
4. Li, Y., and Zamble, D. B. (2009) pH-responsive DNA-binding activity of *Helicobacter pylori* NikR. *Biochemistry* **48**, 2486-2496
5. Micsonai, A., Bulyáki, É., and Kardos, J. (2021) BeStSel: From Secondary Structure Analysis to Protein Fold Prediction by Circular Dichroism Spectroscopy. in *Structural Genomics: General Applications* (Chen, Y. W., and Yiu, C.-P. B. eds.), Springer US, New York, NY. pp 175-189
6. Micsonai, A., Wien, F., Bulyáki, É., Kun, J., Moussong, É., Lee, Y.-H., Goto, Y., Réfrégiers, M., and Kardos, J. (2018) BeStSel: a web server for accurate protein secondary structure prediction and fold recognition from the circular dichroism spectra. *Nucleic Acids Res.* **46**, W315-W322
7. Micsonai, A., Wien, F., Kernya, L., Lee, Y.-H., Goto, Y., Réfrégiers, M., and Kardos, J. (2015) Accurate secondary structure prediction and fold recognition for circular dichroism spectroscopy. *Proc. Natl. Acad. Sci.* **112**, E3095-E3103
